# Development of a Smart Wireless Multisensor Platform for an Optogenetic Brain Implant

**DOI:** 10.3390/s24020575

**Published:** 2024-01-16

**Authors:** André B. Cunha, Christin Schuelke, Alireza Mesri, Simen K. Ruud, Aleksandra Aizenshtadt, Giorgio Ferrari, Arto Heiskanen, Afia Asif, Stephan S. Keller, Tania Ramos-Moreno, Håvard Kalvøy, Alberto Martínez-Serrano, Stefan Krauss, Jenny Emnéus, Marco Sampietro, Ørjan G. Martinsen

**Affiliations:** 1 Department of Physics, University of Oslo, Sem Sælands vei 24, 0371 Oslo, Norway; andre.cunha@fys.uio.no (A.B.C.); christin.schuelke@fys.uio.no (C.S.); skruud@gmail.com (S.K.R.); 2 Hybrid Technology Hub—Centre of Excellence, Institute of Basic Medical Sciences, P.O. Box 1110 Blindern, 0317 Oslo, Norway; aleksandra.aizenshtadt@medisin.uio.no (A.A.); s.j.k.krauss@medisin.uio.no (S.K.); 3 Department of Electronics Information and Bioengineering, Politecnico di Milano, Piazza L. da Vinci 32, 20133 Milan, Italy; alireza.mesri@polimi.it (A.M.); giorgio.ferrari@polimi.it (G.F.); marco.sampietro@polimi.it (M.S.); 4 Department of Biotechnology and Biomedicine, Technical University of Denmark, 2800 Kongens Lyngby, Denmark; arhe@dtu.dk (A.H.); aafiaasif@gmail.com (A.A.); jemn@dtu.dk (J.E.); 5 National Centre for Nano Fabrication and Characterization, Technical University of Denmark, 2800 Kongens Lyngby, Denmark; suke@dtu.dk; 6 Lund Stem Cell Center, Division of Neurosurgery, Department of Clinical Sciences, Faculty of Medicine, Lund University, 22184 Lund, Sweden; tania.ramos-moreno@med.lu.se; 7 Department of Clinical and Biomedical Engineering, Oslo University Hospital, Sognsvannsveien 20, 0372 Oslo, Norway; havard.kalvoy@ous-hf.no; 8 Department of Molecular Neurobiology, Center of Molecular Biology ‘Severo Ochoa’, Universidad Autónoma de Madrid, Calle Nicolás Cabrera 1, 28049 Madrid, Spain; amserrano@cbm.csic.es; 9 Department of Immunology and Transfusion Medicine, Oslo University Hospital, P.O. Box 4950, 0424 Oslo, Norway

**Keywords:** neural stem cells, Parkinson’s disease, dopamine, optogenetics, brain implant, impedance, electrochemistry, amperometry, cyclic voltammetry, PSoC

## Abstract

Implantable cell replacement therapies promise to completely restore the function of neural structures, possibly changing how we currently perceive the onset of neurodegenerative diseases. One of the major clinical hurdles for the routine implementation of stem cell therapies is poor cell retention and survival, demanding the need to better understand these mechanisms while providing precise and scalable approaches to monitor these cell-based therapies in both pre-clinical and clinical scenarios. This poses significant multidisciplinary challenges regarding planning, defining the methodology and requirements, prototyping and different stages of testing. Aiming toward an optogenetic neural stem cell implant controlled by a smart wireless electronic frontend, we show how an iterative development methodology coupled with a modular design philosophy can mitigate some of these challenges. In this study, we present a miniaturized, wireless-controlled, modular multisensor platform with fully interfaced electronics featuring three different modules: an impedance analyzer, a potentiostat and an optical stimulator. We show the application of the platform for electrical impedance spectroscopy-based cell monitoring, optical stimulation to induce dopamine release from optogenetically modified neurons and a potentiostat for cyclic voltammetry and amperometric detection of dopamine release. The multisensor platform is designed to be used as an opto-electric headstage for future in vivo animal experiments.

## 1. Introduction

**Figure 1:**
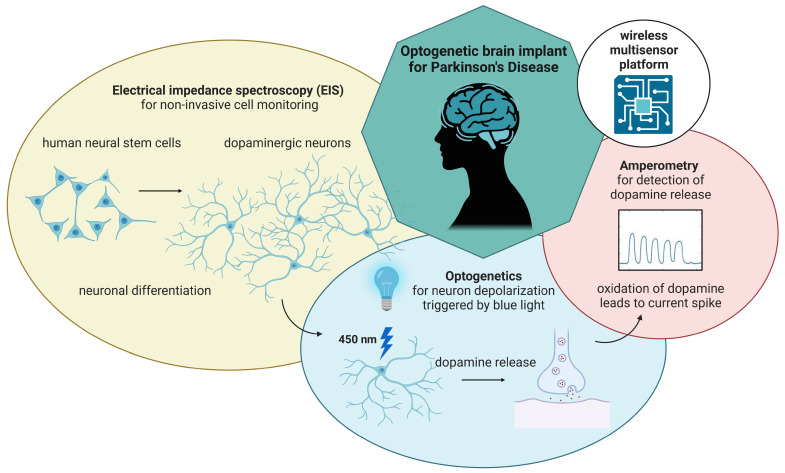
Miniaturized, wireless-controlled, modular multisensor platform designed as an optogenetic brain implant for Parkinson’s Disease. The multisensor platform features three different modules: an impedance analyzer for cell monitoring, an optical stimulator to induce dopamine release from optogenetically modified neurons and a potentiostat for cyclic voltammetry and amperometric detection of dopamine release.

Over the past 30 years, stem cell technologies matured from an attractive option to investigate many diseases to a possible paradigm shift in their treatment through the development of cell-based regenerative medicine (CRM). Implantable cell replacement therapies promise to completely restore the function of neural structures, possibly changing how we currently perceive the onset of these conditions (see Figure 1).

Parkinson’s disease (PD) is a long-term neurodegenerative disorder with a prevalence of 160 per 100,000 people in Western Europe alone, rising to 4% in the population over 80 years [1]. Globally, PD affects around 6.1 million people [2]. Over a period of 7 to 15 years, patients experience worsening motor and non-motor symptoms like a resting tremor, stiffness (rigidity), slowness of movement (bradykinesia), shuffling steps, soft voice, small handwriting (micrographia), postural instability, disturbances of mood, cognition, sleep, and autonomic dysfunction, leading to dementia and death. A high prevalence of cognitive and behavioral problems, such as depression, anxiety and apathy, often correlates with PD, which increases the high emotional, financial and social burden in an increasingly aging population [3]. The pathological hallmark of PD is the dopaminergic cell loss within the substantia nigra pars compacta, which is a critical brain region for the production of dopamine. Dopamine is an important neurotransmitter for the central nervous system, mediating functions such as movement control, cognitive executive functions and emotional limbic activity [4].

While the cause of cell death in PD patients is still a topic of ongoing research, the regeneration of the dopaminergic neurons through CRM in pre-clinical studies involving animal models and human fetal tissue indicates encouraging results. The neurons grafted in the striatum not only survived but also extended axons, which formed synapses with host striatal neurons spontaneously releasing dopamine while reversing some behavioral deficits in animal models of PD [5].

Still, numerous challenges remain until a viable PD CRM treatment will become a standard procedure. In the first CRM treatments for PD in the 1980s, nerve cells derived from fetal tissue were transplanted into the patients’ brain. Despite some successes, only a small number of CRM treatments for PD have reached the clinical trial stage with varying efficacy and graft viability. Reasons for failure can often only be evaluated post-mortem [6]. Lewy body pathology, early degeneration in grafted neurons or maladaptive plasticity in otherwise healthy grafts seem to be some of the medical challenges that suggest the increasing need for close monitoring strategies and new control methodologies for these treatments [5,7,8]. From a cost analysis point of view, CRM treatments are still extremely costly, falling in the million-dollar range. Currently (according to clinicaltrials.gov, accessed on 10 November 2023), a total of five clinical trials involving the transplantation of stem cells for the treatment of PD are being performed or recruiting participants. In Europe, the STEM-PD Trial involves the transplantation of embryonic stem cell-derived dopamine progenitors into the putamen of PD patients [9]. In China, clinical studies involve the transplantation of human mesenchymal stem cells (ClinicalTrials.gov ID NCT03550183, NCT02611167), human amniotic epithelial stem cells (ClinicalTrials.gov ID NCT05691114), and autologous-induced neural stem cell-derived dopaminergic precursor cells (ClinicalTrials.gov ID NCT05901818, accessed on 10 November 2023).

In most cases, Deep Brain Stimulation (DBS) as an alternative highly invasive procedure has been shown to be more effective than CRM in improving patients’ conditions [10]. DBS has thus emerged as a common treatment for PD when pharmacological solutions prove to be or become ineffective. In DBS, a transmitter sends electrical impulses through electrodes implanted in affected brain areas to compensate for the dopaminergic deficit. The electrodes connect to a battery-powered implantable pulse generator (IPG) implanted below the collarbone [11]. Although highly innovative, this technique relies on the remaining neural structures. In a progressive neurodegenerative condition, this treatment becomes ineffective when the condition becomes too severe. Arguably, while it improves the quality of life of patients by mitigating symptoms, it can also speed up neural loss due to cell stress imposed by the electrical stimulation and tissue scarring at the tissue–electrode interface, requiring recurrent highly invasive maintenance procedures which become increasingly ineffective as the condition progresses [12].

Therefore, improved therapeutic strategies for PD are needed. Scientists have now garnered significant experience in developing viable grafts of dopaminergic neurons consistently. Optogenetics via channelrhodopsin (ChR) expression in dopaminergic neurons offers the possibility of precise control over the implanted neurons and therefore over dopamine release. Under light stimulation, channelrhodopsin will change its conformation and open its pore, permitting the influx of cations, thus depolarizing the neurons [13]. The depolarization will trigger the release of neurotransmitters like dopamine that could alleviate the symptoms arising from the lack of dopamine in the brain of PD patients. Vast medical experience from DBS provides a good backbone for surgically similar approaches while offering room for innovation.

While the success of CRM treatments for PD provides the motivation to pursue this line of research, the mixed results regarding the degeneration of the implanted grafts or simply its ineffectiveness suggest a need to control the graft via a multisensor approach. Most cell grafts fail to integrate and survive. A combined multisensor approach could provide us with precious data at different stages without the need for surgery or often challenging imaging techniques.

Live bioimpedance recordings are a promising candidate to monitor those treatments in real time [14]. Impedance means to oppose (=impede) the flow of current. Unlike ohmic resistance, impedance is dependent on the frequency of the applied alternating voltage. Many studies based on impedance measurements of live biological cells enabled the technique to become widely accepted as a label-free, non-invasive and quantitative analytical method to assess cell status. To show some examples, bioimpedance can be used to monitor proliferation, apoptosis, migration, degeneration, morphological changes and also (neuronal) differentiation [15].

Regarding dopamine detection, electrochemical methods are an effective tool to study chemical and biological systems. By measuring the electrochemical reactions that occur when an electroactive analyte such as dopamine interacts with a sensing electrode, it is possible to measure the release of dopamine from living cells and tissues [16]. To perform electrochemical measurements on dopaminergic neurons, a chip with multiple electrodes can be used to act as an interface between cells and an electronics circuit. At least two electrodes are needed to apply an excitation signal to the solution surrounding the cells and measure the resulting current. The measured current will be converted to a voltage signal by a transimpedance amplifier (TIA) and then converted to a digital signal by an analog-to-digital converter (ADC). In a two-electrode system, the working electrode (WE) is the electrode at which the reaction of interest takes place, and the counter electrode (CE) is used to apply the voltage to the sample/system under test (SUT).

Electrochemistry has been used to detect fluctuations in dopamine concentrations in vitro and in vivo with high spatial and temporal resolution. Amperometry and fast-scan cyclic voltammetry are the most prevalent methods for monitoring neurotransmitter release. In amperometry, the WE is held at a constant DC voltage which is sufficient to oxidize or reduce the analyte of interest at the electrode surface. A disadvantage of this technique is the poor specificity, as it does not distinguish any potentially similar electroactive substances present in the SUT. In amperometry, the presence of any other electroactive species that undergo electron transfer at the applied voltage will contribute to the Faradaic current detected at the electrode [17,18].

Cyclic voltammetry (CV) is a powerful technique commonly used to investigate the oxidation and reduction processes of molecular species. In this technique, the voltage applied to the electrode is a triangular waveform, so that molecules are oxidized and reduced repeatedly. Since different molecules oxidize/reduce at different voltages, any other electroactive species present will oxidize/reduce at different times. Therefore, CV allows for some amount of discrimination and identification of the released compounds [17,18,19]. However, it is still not possible to distinguish between similar electroactive substances. Fast-scan cyclic voltammetry (FSCV) utilizes scan rates a thousand times faster than conventional CV. For dopamine, a scan rate of 400 V/s that is repeated at 10 Hz is typically used [20]. FSCV allows for highly sensitive dopamine detection (down to concentrations of 15 nM) and even some possibility of discrimination between different kinds of electroactive molecules by using custom waveforms. However, in FSCV, large amounts of data are generated. The typical scan rate for dopamine of 400 V/s and 10 Hz would generate 36,000 CVs per hour, which demands highly automated data analysis [20]. Furthermore, FSCV requires a larger voltage range that is typically from −0.4 to 1.3 V for dopamine [20], while a constant voltage of only 250 mV is sufficient to detect dopamine by amperometry. FSCV also demands frequently repeated background subtraction. Therefore, amperometry was chosen for the implant, as it has a much lower power demand than FSCV both for generating the signal as well as for storing and analyzing the data.

Recent studies have fabricated different kinds of sensors for dopamine detection, using for example specific surface functionalizations, DNA aptamer-based biosensors, field–effect transistors (FETs) or electrolyte-gated transistors (EGTs), surface-enhanced Raman spectroscopy, or a combination of all of them [21,22,23]. Carbon electrodes can be functionalized with different modifications like nanomaterials from metals or metal oxides, semiconductors, polymers, hydrophilic materials, thiols, enzymes, etc. to increase the detection sensitivity and confer antifouling abilities for long-term stability [22,24,25]. Electrode surfaces can also be functionalized with aptamers. Those are short, single-stranded DNA (or RNA) sequences, typically 25–70 nucleotides long, that can bind a particular molecule by folding around the interaction partner in a specific 3D structure by adaptive conformational change through hydrogen bonds and electrostatic interactions [26]. Similar to antibodies, aptamers can bind to a target with high affinity and can be optimized to have a high specificity [27]. DNA aptamers specific for dopamine have been engineered and incorporated into biosensors for in vitro and in vivo dopamine detection [28,29,30].

In addition to electrochemical sensors, field-effect transistors (FET) are a different group of biosensors typically utilized for small molecule detection. FETs are mainly composed of three electrodes (gate, source and drain), an insulating layer and a semiconductor layer. If a target analyte binds to the FET, charged ions are generated, which will further induce the change of carriers in the channel material [31]. Electrolyte-gated transistors (EGTs) are a modified version of FETs, where the traditional insulating layer material was replaced by electrolytes, such as polymers or ionic liquids [31].

Those improvements provide great sensitivity, higher specificity, possibility for miniaturization, and enhanced electrode stability but often require complex fabrication processes and surface functionalization with usually organic chemicals that are difficult to implement for an in vivo biosensor. Most biosensors were only tested in vitro so far and are often not stable in complex in vivo environments. On the contrary, Abrantes et al. fabricated micron-sized electrolyte-gated field-effect graphene transistors (EG–gFETs) functionalized with dopamine-specific DNA aptamers for ultrasensitive dopamine detection, where the limit of detection was as low as 1 aM (10^−18^ M) [32]. They were able to detect dopamine in brain homogenates and cerebral spinal fluid samples obtained from a mouse model of Parkinson’s disease.

Although impedance and amperometry are considered non-specific measurements, together they provide a rather complete picture for complex but well-characterized samples. These methods have been used for characterization in fairly predictable environments for a long time. By performing synthetic, in vitro and in vivo pre-clinical studies, it is reasonable to expect that we will eventually be able to characterize the biological SUT with enough detail. This is especially true considering that state-of-the-art DBS is mostly a blind treatment with no live monitoring during the patient’s life. Although recent studies have developed a similar approach [33], to the best of our knowledge, we are the first to fully integrate an in vitro study combining impedance and electrochemical methods.

In this work, we present a multisensor platform using fully integrated modules that enable both impedance spectroscopy to monitor cell growth as well as optical stimulation and electrochemical measurements to test the performance of optogenetically modified dopaminergic neurons. In the first phase of the study, we monitored cell growth using impedance measurements. After the cells were differentiated into dopaminergic neurons, we stimulated them optically and performed amperometric measurements to monitor dopamine release. In future studies, we plan to interface this platform, which is designed to be an opto-electric headstage, with a cell grafted leaky opto-electrical fiber [34,35] that has been developed in parallel in the Training4CRM consortium (European Training Network for Cell-based Regenerative Medicine) to proceed to in vivo studies. The use of both impedance and electrochemical measurements provides a more comprehensive understanding of the performance of dopaminergic neurons and can aid in the development and optimization of cell-based therapies for PD and other dopamine-related disorders.

## 2. Materials and Methods

### 2.1. Overview

The developed multisensor platform is designed to be interfaced with a specially developed leaky opto-electrical fiber [34,35]. This fiber is to be grafted with optogenetically modified neural progenitor cells. These cells grown on the fiber are differentiated to dopaminergic neurons. The multisensor platform relies on two different sensing technologies, which focus on different clinical objectives. After implantation, it is important to periodically assess the health of the implant regarding structural changes of the cell tissue related to common issues with stem cells such as teratoma or abnormal cell apoptosis. For this purpose, the developed multisensor platform utilizes non-invasive impedance spectroscopy measurements. Impedance measurements can assess cell growth and cell differentiation but do not provide any information about the effectiveness of both the optogenetic stimulation nor the release of dopamine from the cells [15]. For this purpose, the sensor utilizes a potentiostat implementation, which is a proven and accurate method to measure dopamine levels even in free moving animals [36].

To minimize study variables and to facilitate in vitro measurements, a previously studied in vitro cell culture and electrode system was used as SUT. The specially developed leaky opto-electrical fiber was studied separately using the same cell line [34,35]. The full integration of the leaky opto-electrical fiber with the electronic interface will be approached in future studies.

In this work, we do not only describe each sensor and actuator component but actually focus on an application-driven study. For the impedance analyzer, we study its performance in a cell growth experiment by comparing it to a standard state of the art lock-in-amplifier based desktop impedance analyzer, a Zurich Instruments MFIA (Zurich Instruments, Zürich, Switzerland). For the potentiostat, we perform cyclic voltammetry measurements using solutions of ferri-/ferrocyanide and dopamine followed by the study of dopamine release from neurons differentiated from optogenetically modified neural progenitor cells. This application-oriented approach shows the ability of the device to perform impedimetric and amperometric cell measurements.

### 2.2. Electronic Instrumentation

**Figure 2:**
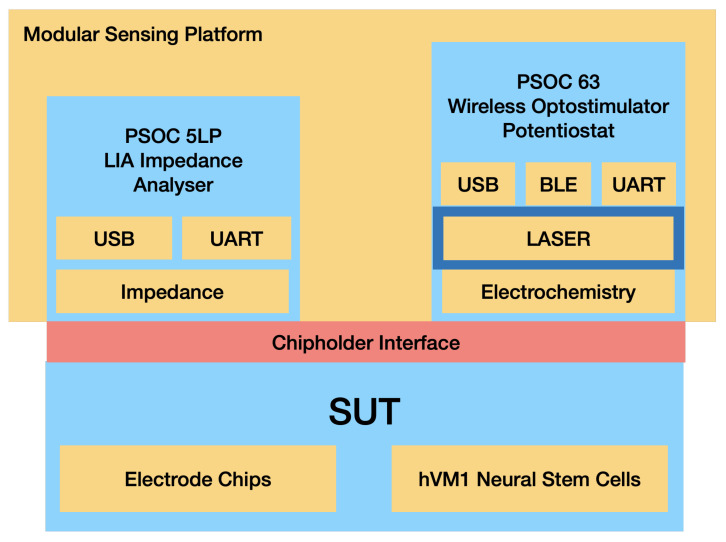
Schematic for the Optogenetic Multisensor Platform Modules.

The PSoC™ (Programmable System on Chip) development platform (Infineon Technologies, Neubiberg, Germany) is a flexible, compact and versatile platform that allows for easy customization and development. By abstracting hardware development into a common software development platform of programmable analog and digital blocks, the PSoC™ platform simplifies the process of designing and building sensor systems. The use of this platform in the multisensor platform allows for the development of a modular system that can be easily adapted to different clinical scenarios. To fully exploit this feature of the PSoC™ development platform, the sensor’s electronic headstage was designed as a modular stacked unit with interchangeable parts for different clinical scenarios. Conceptually, the developed multisensor platform consists of three different modules: an impedance analyzer, a potentiostat and an optical stimulator. The PSoC™ platform MCU controls all the modules, and communication relies on an embedded BLE (Bluetooth low energy) unit and USB (see Figure 2).

These units have been developed in parallel, and some proof-of-concept performance studies have been performed before [37]. In this paper, we show a pre-clinical application focused on impedance and electrochemical measurements on in vitro cell cultures and respective control measurements. This paper not only integrates the modules developed under Training4CRM but also improves aspects on all of them while showing its performance in an integrated laboratory study.

#### 2.2.1. Optical Stimulator

The optical stimulation headstage allows a controlled release of dopamine by the optogenetically modified neurons. An OSRAM (Munich, Germany) blue laser diode (LD), PL 450B, was selected as a light source for the system. The main features of this LD are 450 nm emission wavelength, 3.8 mm package size, and 80 mW optical power. With 40 mA driving current at 20 °C, it can provide about 25 mW optical power. A GPIO pin (General Purpose Input Output) provides the stimulation pattern in the form of a pulse width modulation (PWM) signal for the LD driver chip. This will be converted to a DC voltage by a low-pass filter before applying it to the LD driver. Another GPIO pin is a shutdown (SHDN) signal, which turns on the LD driver chip during the optical stimulation. The maximum available current at the LD pin is defined by an off-chip resistor ( $R_{SET}$ ).

Linear Technology (Milpitas, CA, USA) LT1932 is a constant frequency step-up DC/DC converter that works as a constant-current source and drives the LD. The LD current can be from 5 to 40 mA, and the value of $R_{SET}$ defines the current value. There are different ways to change the value of the LD current. Changing the LD pin current, the filtered PWM signal is applied to the $R_{SET}$ pin. In this method, due to the internal structure of LT1932, decreasing the duty cycle of the PWM signal will increase the LD current and thus its optical power. The PWM signal is generated directly by the BLE SoC, and its duty cycle can be easily controlled through the firmware to adjust optical power. Therefore, there is no need for additional circuits to set the current of the LD.

The PCB (printed circuit board) of the optical stimulator also carries two Zinc/monovalent Silver Oxide SR927W batteries (Renata, Itingen, Switzerland), which are used to provide the supply voltage to the entire headstage. They have a capacity of 55 mAh and a nominal voltage of 1.55 V. Their weight is about 0.77 g each.

**Figure A1:**
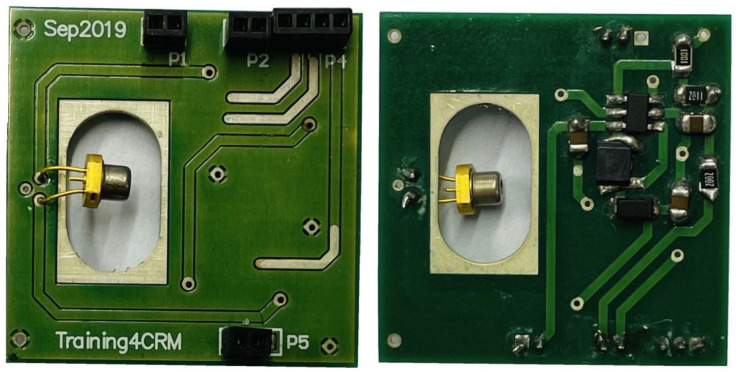
Optical stimulator board.

The performance for the optical stimulator circuit has been studied in detail in a previous iteration by using a specially built testing card based on the Anaren (East Syracuse, NY, USA) A20737 BLE SoC [38]. In this work, the optical stimulator is coupled to the potentiostat module, as described further, with the purpose of performing optogenetic stimulation experiments with dopamine detection. The optical stimulator board is depicted in Figure A1.

In this work however, instead of relying on the CPU to generate the optical signal, the optical pattern is determined by a fixed logic circuit using the digital elements provided by the PSoC™ that can be configured similarly to an FPGA. For programming a pulse train, we use two timer counters that modulate the small pulses and the total pulse duration. The PWM controls the LD power output and is controlled by both timers together (see Figure A8). This means that after it is programmed, the stimulation pattern is fixed, although timings can be adjusted by setting different clock settings. It operates with no CPU intervention, saving both power and computational resources. To change it, the chip needs to be reprogrammed, which can be completed over the air (OTA), as PSoC™ 63 supports this feature.

#### 2.2.2. Impedance Analyzer

Advances in portable sensing systems and smart sensor interfaces enabled the monitoring of complex biomedical environments. One particular challenge in the brain, adding to the general challenges for any biological environment, is the presence of large amounts of electrical noise resulting from brain activity, whose sources are diverse but the result is always the same: the contamination of sensor output signals [39]. A state-of-the-art approach to solve this issue is the lock-in amplifier (LIA), which uses a phase-sensitive detection (PSD) to filter out the data signal at a specific reference frequency and to reject noise signals at other frequencies without affecting the measurement significantly.

In this work, we propose a digital LIA implementation which exploits the PSoC™ 5LP unique architecture enabling us to combine both analog and digital elements (see Figure A5 and Figure A6). By using mostly embedded components for the LIA, we expect to significantly reduce noise and avoid interfacing challenges from using different components while retaining high miniaturization as good as an ASIC (application-specific integrated circuit) implementation.

**Figure 3:**
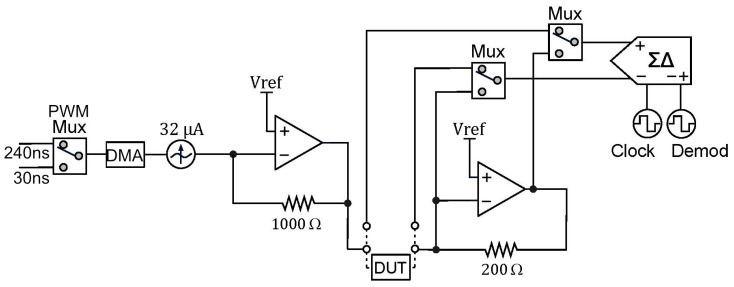
Schematic for the DLIA (digital lock-in amplifier) implementation used for impedance measurements on the developed multisensor platform. Apart from passive components, the implementation was prototyped in a PSoC™ (Programmable System on Chip) 5LP Development Kit. Logic systems to control sine wave excitation signals, the state machines to operate the ADC (analog-to-digital converter) between the reference and measured signal and also polarity demodulation control and LUT tables are available in the Appendix A.

Our proposed instrumentation for impedance measurements based on a DLIA implementation is detailed in Figure 3. By switching the polarity of the ADC using PSoC™, we multiply the sinusoidal input signal by ±1. To the best of our knowledge, this is only possible in the PSoC™ architecture where the programmable mixed signal arrays can be controlled to operate the embedded ADC (PSoC™ Creator Component Datasheet Delta Sigma Analog to Digital Converter ADC_DelSig 3.0) in this manner. By doing this, we eliminate any DC offset noise component that would arise by multiplying by 0 and 1 in a grounded square wave. To deal with additional noise from harmonics that occurs from square wave multiplication, we simply use a 20-bit delta-sigma ADC that widely oversamples the signal and low-pass filters it. Neither anti-aliasing nor a sample and hold circuit are required.

Both in-phase and quadrature components are measured and calculated. The integrated PSoC™ architecture allows for generating both excitation signals using the same source. We trigger both measurements by using a finite state machine which alternates from both components. For increased precision, the excitation signal is also measured internally by using an internal switching system to acquire the signal directly. In the end, all measurements are used in the MCU to perform standard calculation of both in-phase and quadrature components as described in Figure A7.

In a previous report, we were able to obtain promising preliminary results [37]. In this iteration, we improved the circuit to make use of a dual PWM (pulse width modulation) system to significantly expand the frequency range from previously 10 Hz to 100 kHz to currently 10 Hz to 300 kHz with a 32 mV sine wave excitation signal.

The current source is a DAC (digital-to-analog converter) providing a signal with a constant alternating current set to 31.875 µA, which is the lowest of the three options available. To provide an alternating current, the DAC sources current from voltage sources both above and below its output voltage. In the case of the PSoC™, it does not have voltage references below ground, and therefore, biasing the signal with a DC voltage is needed. This is accomplished by converting the output current of the DAC into a voltage using a transimpedance amplifier with the non-inverting node at VDDA/2. While this could be identified as an issue, the transimpedance amplifier for the current readout is also referred to VDDA/2, and as far as the SUT is concerned, there is no DC offset. The output current of the DAC is converted to a voltage using a 1000 Ω resistor, resulting in an excitation signal amplitude of 32 mV .

The DAC is in reality strictly outputting a current based on its polarity and given 8-bit value. To provide the signal with a waveform and a given frequency, we control the timing of DMA (direct memory access) transactions by using PWMs. With the 26-point sine wave used in this configuration, the same amount of DMA transactions would correspond to one period of the excitation signal. Using a single PWM with the bus clock would not provide low enough frequencies because of its 16-bit resolution. By adding a second PWM with a lower clock reference, we could overcome this limit. A digital multiplexer with a control register was added to switch between the two PWMs.

The Delta-Sigma ADC is configured for 20-bit resolution, ±0.256 V input range, and the sampling rate is controlled by the input clock, which can be set in runtime. The chosen input range allows for a minimum of 2 and maximum of 15 samples per second and a clock rate of approximately 1 MHz. When measuring low-frequency signals, it is important to lower the rate, as a sample should cover a number of periods in the signal. A minimum of ten periods has been the reference in this design.

The measured signal is modulated and averaged, and the end result is a value that would be equal to the peak amplitude of a sine wave multiplied by 2 π . Separate in-phase and in-quadrature components are measured through using the modulation input clock, which is controlled by a digital circuit. This modulation clock implements phase detection in the ADC by periodically inverting the analog input of the ADC and as a result functions as a type of square wave lock-in amplifier.

Demodulation with a pure sine wave enables selective measurement at the fundamental frequency or any of its harmonics. This instrument uses a square wave which also captures all odd harmonics of the signal and therefore potentially introduces systematic measurement errors. However, the systematic errors are also strongly reduced by using a sinusoidal stimulus. Since the SUT is expected to have a predominantly linear response, a square wave demodulation should not add errors, because at the input, there is only the fundamental frequency.

By both using a delta-sigma ADC in continuous mode, highly oversampling the measurements but also by relying on relative impedance measurements to assess cell growth and possibly differentiation, these systematic errors are easily removed, as they should be present in the reference measurement, and they are also related to the excitation signal itself and not so much to the SUT.

#### 2.2.3. Potentiostat

A potentiostat is an electronic instrument to perform electrochemical measurements. In this work, we use a three-electrode system. In a three-electrode system, the potential change of the WE is measured independently of changes that may occur at the CE. An additional electrode, called the reference electrode (RE), is added to the setup. It works as a non-current conducting electrode, acting as a reference in controlling and measuring the voltage of the electrolyte.

We use a potentiostat to perform amperometry and cyclic voltammetry (CV), which are the most prevalent methods for monitoring neurotransmitter release. In amperometry, the voltage is held at a DC voltage sufficient to oxidize or reduce the analyte of interest at the electrode surface. In CV, the applied voltage is a triangular waveform, and the voltage limits are chosen so that the oxidation and reduction of the compound of interest lie within this voltage window.

The DAC generates a constant voltage for amperometry and a triangular waveform for CV. The TIA, which is connected to the WE, converts the measured current into voltage in order to be converted to a digital signal by an ADC. The ADC values are then computed in the MCU (microcontroller unit) to calculate the measured signal as a function of time or of the applied voltage for amperometry and voltammetry, respectively [40]. A differential amplifier senses the voltage of the RE and compares it with the DAC output. The amplifier drives the CE electrode in order to have the RE at the desired voltage selected by the DAC irrespective of the current flowing in the system. In this way, a much better control of the experiment is obtained.

**Figure 4:**
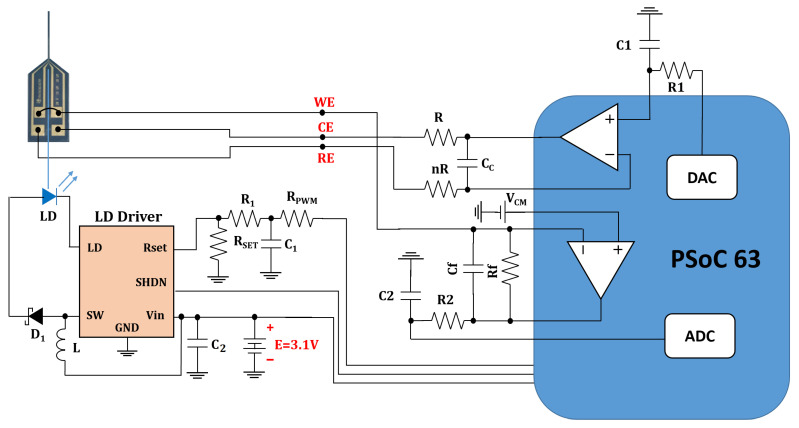
Schematic for full opto-electrochemical platform including the optical stimulation module and the electrochemical sensing module for cyclic voltammetry and amperometry. As shown in the schematic, the ADC (analog-to-digital converter), DAC (digital-to-analog converter) and opamps (operational amplifier) are embedded components of the PSoC™ 63 chip used in the prototype board for the electrochemical sensing module.

**Figure 5:**
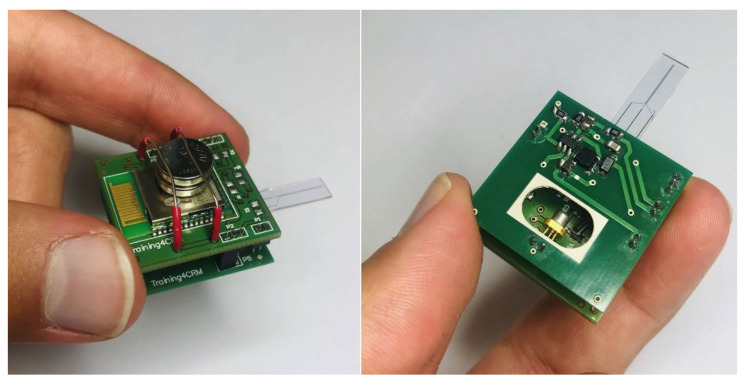
Fully assembled hardware prototype for optical stimulation and electrochemical sensing. Pictures of individual components available in the Appendix A.

**Figure A2:**
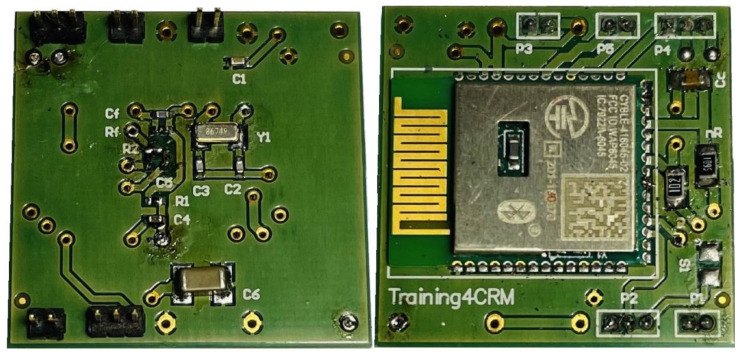
Electrochemical sensing board.

**Figure A5:**
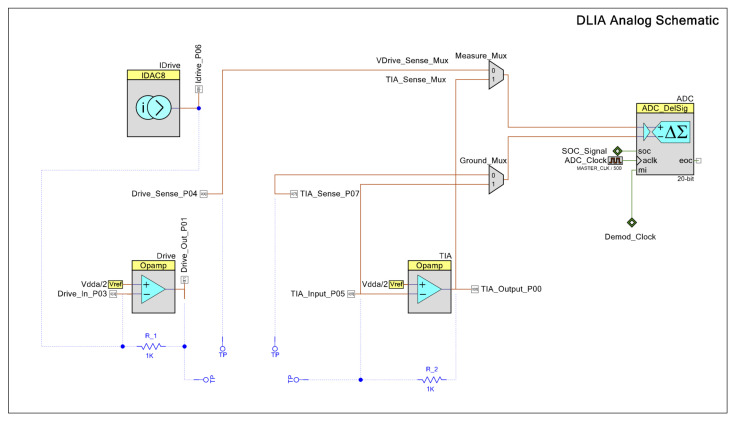
DLIA implementation schematic. PSoC™ Creator schematic: Analog PSoC™ Creator embedded components shown above.

**Figure A6:**
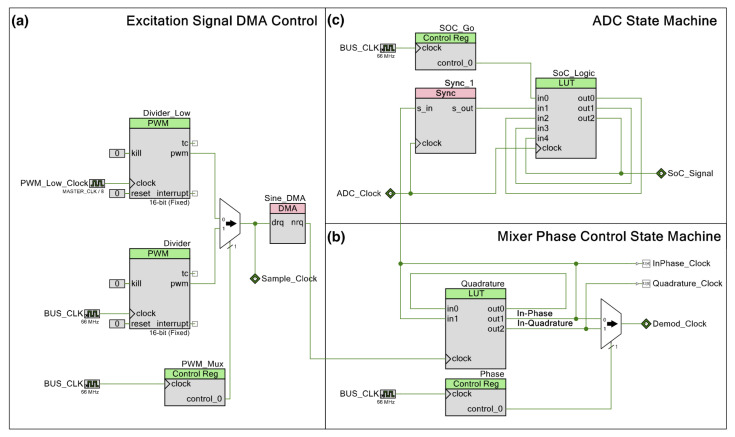
DLIA logic system schematic. PSoC™ Creator schematic: Digital PSoC™ Creator embedded components shown above. (**a**) The PWM generates the clock which triggers the DMA transactions. The frequency of the waveform generation can be changed by adjusting the period of the dividers every quarter wave, trigger NRq, every half wave, change sign of IDAC. (**b**) This state machine generates the in-phase and in-quadrature clocks for the multiplying up-mixer. The phase control register is used to select which clock is sent to the mixer. (**c**) This state machine generates the start of conversion (SOC) signal for the ADC. It is armed by setting the bit in the SOC_Go control register. On the next rising edge of the in-phase clock, the SOC signal will be generated.

**Figure A7:**
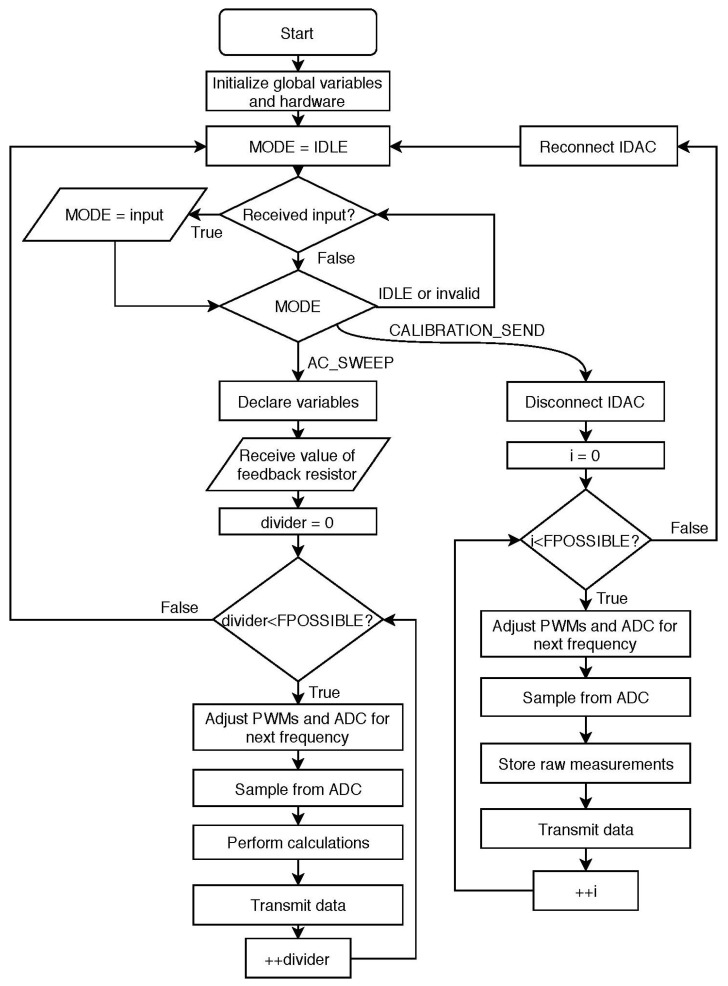
DLIA firmware flowchart. Code available upon request.

**Figure A8:**
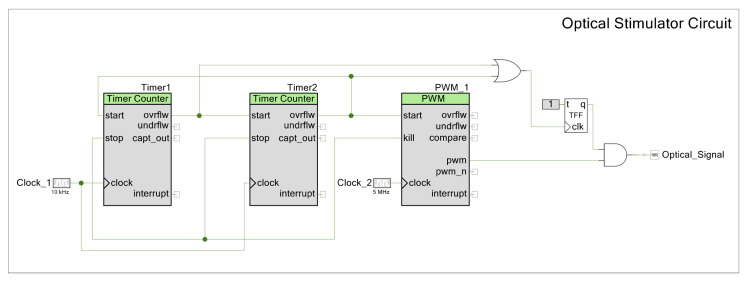
Laser stimulator signal logic schematic. Digital PSoC™ Creator embedded components shown above. The two timers modulate the pulse train. The fast timer modulates the pulse duration of each train and the slow timer modulates how much time passes between each pulse train. This is permitted by the overflow of each timer. The PWM defines the laser pulsing within each pulse of the pulse train. The additional logic and the flip-flop combined modulate the signal as desired.

Figure 4 shows the schematic of our headstage potentiostat and optical stimulator to perform experiments on optogenetically modified dopaminergic cells. The board itself is designed for an optical stimulation circuit (Figure A1) which connects to the electrochemical sensing platform (Figure A2) based on the PSoC™ 63 chip. The fully assembled hardware prototype for optical stimulation and electrochemical sensing is depicted in Figure 5.

The weight of the optical stimulation board is 1.37 g . Two two-layer boards are used to design the required PCBs. The PSoC™ 63 chip hosts an electrode chip which can be connected through pins to the bottom board, and electrodes are connected to PSoC™ 63 on the top board through a connector. The PSoC™ 63 chip sends the stimulation signal to the LD driver circuit through a connector that connects the top and bottom boards. The total weight of the opto-electrochemical platform is 6.4 g , where 1.5 g comes from the batteries. The dimensions are 26 × 26 × 19 mm , including batteries. These values are compatible with in vivo measurements, wherein the platform is mounted on the head of a mouse or rat. For measurements in mice, the weight and size would still be too large, but we are working on further miniaturizing the dimensions. The platform is able to send the measured signal or receive instructions through wired or wireless communication.

#### 2.2.4. Communications and Control

All communications and control functions are handled by the PSoC™. For optoelectrochemical sensing, wireless was a requirement as the subject should actually be able to move freely as dopamine stimulation takes place. For this purpose, PSoC™ 63 was used. This chip includes both a SoC and a BLE wireless module capable of performing any required calculations and also wireless data transmission.

For this in vitro study and for control reasons, we chose to perform USB (PSoC™ Creator USBFS API component) wired measurements only in order to have better control.

Both systems output data into an UART (universal asynchronous receiver/transmitter, PSoC™ Creator USB-UART API component). In wireless BLE, an UART BLE bridge is used by exploiting the BLE notification system, which can be adapted to act as a bridge for UART if paired with a dedicated BLE receiver seamlessly and automatically. The BLE module automatically searches for a specific receiver with a certain MAC address. The UART-BLE bridge relies on a specific receiver with this implementation, which can be encrypted for privacy and security reasons, although this was not implemented in this study. The UART-BLE bridge is based on the code example CE222046—PSoC™ 6 BLE Throughput Measurement and the UART-BLE bridge for PSoC™ 6, both provided by Infineon.

Calculations related to impedance, electrochemistry and also the control functions described above were all implemented for this work in both the impedance analyzer and the potentiostat. However, given that each of these studies took place in parallel and also for experiment control reasons, in the case of the potentiostat, the wireless communication protocol based on BLE was tested synthetically and separately by connecting the excitation signal directly to the ADC and transmitting the data between two PSoC™ 6 development kits, as shown on the code example CE222046—PSoC™ 6 BLE Throughput Measurement provided by Infineon. PSoC™ 5LP does not possess embedded wireless capabilities, and for the pre-clinical scenarios presented, it is no priority. In a future in vivo study, full integration is required to assess the viability of the platform as a clinical device.

### 2.3. System under Test (SUT)

**Figure A9:**
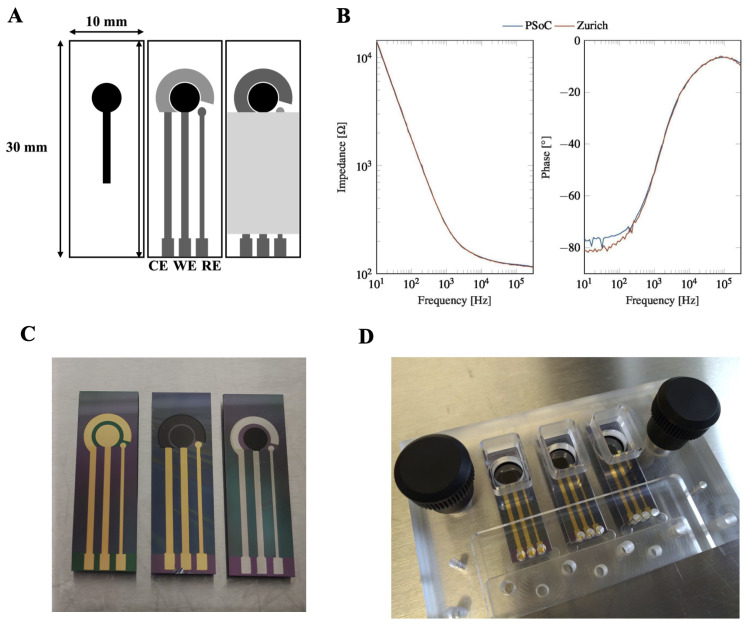
(**A**) Schematic drawing of 2D carbon electrodes: layer of pyrolytic carbon (**first**), platinum electrodes, contact leads and pads (**second**), SU-8 passivation layer (**third**), modified after [43]. (**B**) Impedance and phase angle against frequency for the electrodes using both the proposed instrument based on PSoC™ and the Zurich Instruments MFIA. (**C**) Cleanroom-fabricated electrode chips on silicon wafer substrate with gold WE, CE and RE (**left**), carbon WE and CE and gold RE (**middle**) and carbon WE and platinum CE and RE (**right**). (**D**) Photo of electrodes assembled in micromilled PMMA chip holder.

Impedimetric, electrochemical and optogenetic measurements with the multisensor platform were performed on human neurons differentiated from neural progenitor cells grown on cleanroom-fabricated 2D electrode chips shown in Figure A9.

#### 2.3.1. Cell Culture

The optogenetically modified human fetal ventral mesencephalic neural progenitor cell line hVM1-Bcl-XL-GFP-ChR2-mCh was used in this study. The hVM1 cell line was established by isolating human neural progenitor cells from a fetal ventral midbrain and immortalized by stable transfection with v-myc [41]. Villa et al. have shown that neuronal differentiation results predominantly in tyrosine hydroxylase-expressing neurons. Differentiation to dopaminergic neurons was improved by stable transfection with the anti-apoptotic protein Bcl-XL (basal cell lymphoma-extra-large) [42]. The cell line was optogenetically modified to express Channelrhodopsin-2 (ChR2) under control of the human synapsin 1 gene promoter by lentiviral transfection with Syn1-ChR2(H134R)-mCherry-WPRE [34]. Cells were routinely cultured on cell culture plasticware coated with Geltrex™ (ThermoFisher Scientific, Waltham, MA, USA, A1413301) in growth medium (GM) saturated with 5% ${\mathrm{CO}}_{2}$ at 37 °C.

Basic Medium (BM): Dulbecco’s modified Eagle medium/F-12 medium with Glutamax (ThermoFisher Scientific, Waltham, MA, USA, Cat 31331028) supplemented with 0.5% Albumax I (ThermoFisher Scientific, Waltham, MA, USA, Cat 11020039), 5 mM HEPES (ThermoFisher Scientific, Waltham, MA, USA, Cat 15630056), 0.6% glucose (Sigma, Merck, Darmstadt, Germany, BioReagent, Cat G7021), and 1% penicillin/streptomycin (Sigma, Merck, Darmstadt, Germany, Cat P4333).

Growth Medium (GM): BM supplemented with N2 supplement (ThermoFisher Scientific, Waltham, MA, USA, Cat 17502048), non-essential amino acids (Ala, Asn, Asp, Glu, Pro; 40 mM each; MerckMillipore, Merck, Darmstadt, Germany, Cat 101007, 101565, 100126, 100291, 107434), 20 ng/mL epidermal growth factor (EGF) and 20 ng/mL basic fibroblast growth factor (bFGF/FGF2, R&D Systems, Minneapolis, MN, USA, Cat 236-EG-200, 233-FB-025).

Differentiation Medium (DM): BM supplemented with N2 supplement, non-essential amino acids, 2 ng/mL glial-derived neurotrophic factor (GDNF, Peprotech, ThermoFisher Scientific, Waltham, MA, USA, Cat 450-10) and 1 mM db-cAMP (N^6^,2′-O-Dibutyryladenosine 3′,5′-cyclic monophosphate sodium salt, Sigma, Merck, Darmstadt, Germany, Cat D0627-250MG).

#### 2.3.2. Neuronal Differentiation

**Figure 6:**
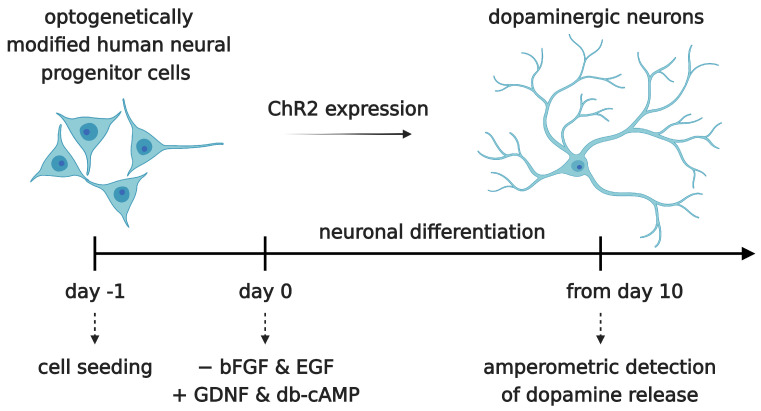
Neuronal differentiation of the optogenetically modified neural progenitor cell line hVM1-Bcl-XL-GFP-ChR2-mCh. Cells were seeded at day -1. At day 0, neuronal differentiation was induced by withdrawal of epidermal growth factor (EGF) and basic fibroblast growth factor (bFGF/FGF2) and the addition of glial-derived neurotrophic factor (GDNF) and db-cAMP (N^6^,2′-O-Dibutyryladenosine 3′,5′-cyclic monophosphate). After a differentiation time of at least 10 days, dopamine release was measured by amperometry.

Neural progenitor cells were seeded onto 2D electrode chips for impedimetric and electrochemical/optogenetic measurements or on coverslips for immunocytochemistry in GM at a density of 30,000 cells/cm^2^. All GM was replaced by DM 24 h after seeding. This is denoted as differentiation day 0 (DD0). Then, 48 h after seeding, all DM was replaced with fresh DM (DD1). Subsequently, 2/3 of the medium was replaced with fresh DM every second day (DD3, DD5, DD7, etc.). The timeline of neuronal differentiation is shown in Figure 6.

#### 2.3.3. Immunocytochemistry

To assess the expression of different marker proteins, cells grown on coverslips were fixed in 4% (*w*/*v*) paraformaldehyde for 15 min at room temperature and washed with PBS three times. Cells were analyzed at different time points before and during differentiation (DD0, DD5, DD15 and DD25). Coverslips were stored in PBS at 4 °C until staining. Cell permeabilization and blocking was performed for 1 h using 10% FBS (*vol*/*vol*) and 0.1% Triton X-100 (*vol*/*vol*) in PBS at room temperature. Cells were washed twice in PBS and incubated in primary antibody solution overnight at 4 °C. Antibody dilution buffer contained 5% FBS (*vol*/*vol*) and 0.05% Triton X-100 (*vol*/*vol*) in PBS. The following primary antibodies were used in this study: NF-H (neurofilament heavy, 1:4000, chicken polyclonal, Biolegend, San Diego, CA, USA, Cat 822601); MAP-2 (microtubule-associated protein 2, 1:1000, rabbit polyclonal, MerckMillipore, Merck, Darmstadt, Germany, Cat AB5622); GFAP (glial fibrillary acidic protein, 1:1000, rabbit polyclonal, Abcam, Cambridge, UK, Cat ab7260); TH (tyrosine hydroxylase, 1:200, mouse monoclonal, Sigma, Merck, Darmstadt, Germany, Cat T1299); NeuN (Neuronal Nuclear Antigen, 1:500, rabbit monoclonal, Abcam, Cambridge, UK, Cat ab177487); and Nestin (1:200, mouse monoclonal, MerckMillipore, Merck, Darmstadt, Germany, Cat MAB5326). Following primary antibody incubation, samples were washed with PBS three times for 15 min each and incubated in secondary antibody, which was diluted in dilution buffer, for 1 h at room temperature. The following secondary antibodies were used: Alexa Fluor 488 goat anti-rabbit (Life Technologies, ThermoFisher Scientific, Waltham, MA, USA, Cat A11008, 1:200); Alexa Fluor 594 goat anti-chicken (Invitrogen, ThermoFisher Scientific, Waltham, MA, USA, Cat A11042, 1:400) and Alexa Fluor 647 donkey anti-mouse (Jackson Immuno Research, West Grove, PA, USA, Cat 715-605-150, 1:400). Nuclei were stained using Hoechst 33258 diluted 1:500 in PBS for 10 min at room temperature, which was followed by washing three times in PBS for 3 min each. Coverslips were mounted on glass slides using gelatin-based mounting medium. Images were acquired using the Laser-scanning confocal microscope Zeiss LSM 700 (Zeiss, Oberkochen, Germany). Images were analyzed using FIJI/ImageJ (US National Institutes of Health, Bethesda, MD, USA, version 1.54f).

#### 2.3.4. Electrodes and In Vitro System

The electrodes used in this study were fabricated in the clean room facility at DTU Nanolab, National Centre for Nano Fabrication and Characterization, Technical University of Denmark and shown in Figure A9. The electrodes feature an on-chip working (WE), counter (CE) and reference electrode (RE). Different electrode material combinations were used, e.g., carbon WE and platinum CE and RE/carbon WE and CE and gold RE/gold WE, CE and RE (see Figure A9). Electrode fabrication is described in [43].

Prior to cell seeding, electrode chips were treated with oxygen plasma to ensure a clean surface and improved ionic conduction and hydrophilicity, as the carbon surface without plasma treatment is too hydrophobic for cell adhesion. Electrodes were then assembled in a micromilled PMMA chip holder shown in Figure A9 in a laminar flow cabinet after all parts of the chip holder had been cleaned using 70% ethanol. The well for cell culturing was sterilized with 0.5 M NaOH solution for 15 min and rinsed with PBS three times. The electrode surface was coated with Geltrex™ (ThermoFisher Scientific, Waltham, MA, USA, A1413301) for at least one hour or preferably overnight to allow for cell adhesion.

#### 2.3.5. Amperometric Detection of Neurotransmitter Release

Cells differentiated to neurons on-chip for at least 14 days were used for the electrochemical detection of neurotransmitter exocytosis. Cell medium was replaced by a physiological buffer containing a low K^+^ concentration (10 mM HEPES, 5 mM glucose, 1.2 mM MgCl_2_, 2 mM CaCl_2_, 150 mM NaCl, and 5 mM KCl, ThermoFisher Scientific, Waltham, MA, USA and Sigma, Merck, Darmstadt, Germany). Exocytosis of neurotransmitters was either triggered by depolarizing the cell membrane by injecting a high K^+^ buffer extracellularly to the cell culture well (10 mM HEPES, 5 mM glucose, 1.2 mM MgCl_2_, 2 mM CaCl_2_, 5 mM NaCl, and 450 mM KCl) or by optical stimulation of the optogenetically modified cells with blue light (450 nm). The electrode chips used in this study had a much larger working area than the leaky opto-electrical fiber [34,35] that was planned to be interfaced with the headstage platform. Specifications of the system were defined and a suitable laser diode was chosen based on the leaky opto-electrical fiber.

Therefore, the power of the laser diode integrated to the platform was not sufficient to stimulate the cells, and an external laser had to be used for optical stimulation (RLD D450-40-5, 450 nm, 40 mW, focusable, <170 mA, Ø 5mm, Roithner Lasertechnik, Vienna, Austria). Amperometry data were analyzed using Matlab 2020 (Portola Valley, CA, USA), Origin 2022 (OriginLab Corporation, Northampton, MA, USA) and filtered using the Savitzky-Golay filter [44] with suitable parameters. A commercial potentiostat (CHI1010A, CH Instruments, Austin, TX, USA) was used for reference measurements.

## 3. Results

### 3.1. Neural Progenitor Cell Growth and Differentiation

Optogenetically modified neural progenitor cells (NPCs) (human fetal ventral mesencephalic neural progenitor cell line hVM1-Bcl-XL-GFP-ChR2-mCh) were differentiated to neurons. The success of neuronal differentiation was assessed by analyzing the expression of different neural and neuronal marker proteins by immunocytochemistry. The differentiation of NPCs leads to neuron-like morphology with neurite outgrowth, smaller cell bodies and the expression of neuronal marker proteins. The following marker proteins were examined: Nestin, NF-H (neurofilament heavy), MAP-2 (microtubule-associated protein 2), GFAP (glial fibrillary acidic protein), TH (tyrosine hydroxylase), and NeuN (Neuronal Nuclear Antigen).

**Figure 7:**
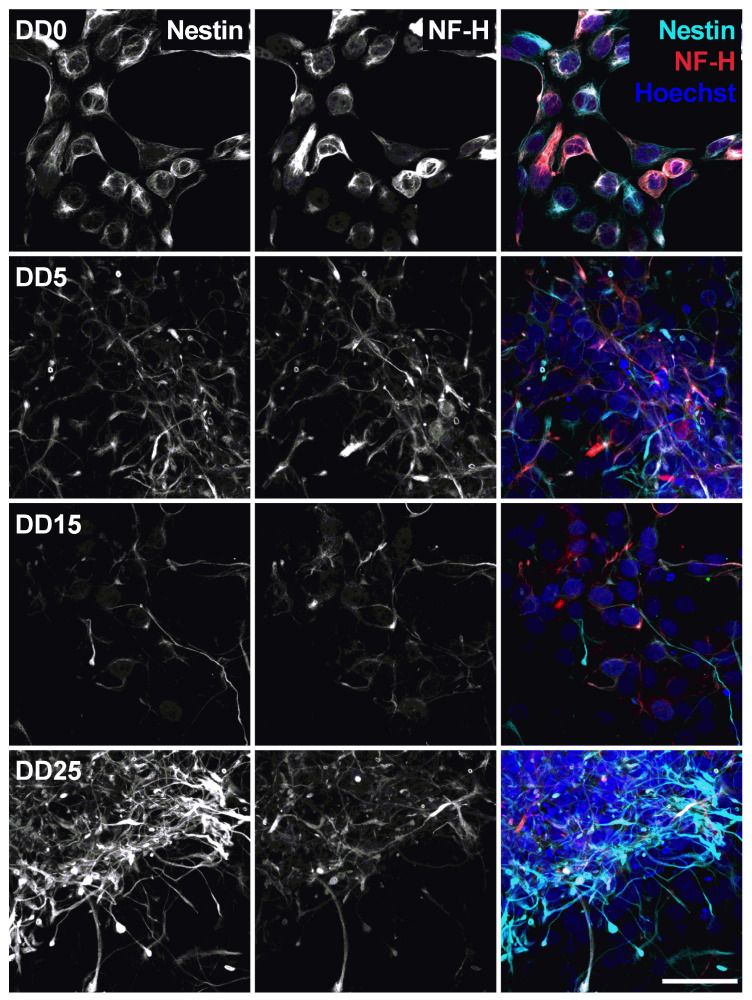
Expression of the neural stem cell marker Nestin and the neurofilament marker NF-H before (differentiation day DD0) and during neuronal differentiation (DD5, DD15 and DD25) of neural progenitor cells. Nuclei were counterstained with Hoechst. Scale bar corresponds to 50 μ m.

Neural progenitors strongly express Nestin and NF-H, as shown in Figure 7. Nestin (neuroepithelial stem cell protein) belongs to the group of intermediate filament proteins that are expressed in the cytoskeleton of proliferative neural stem and progenitor cells and developing neurons [45,46]. Nestin expression is usually downregulated during neuronal differentiation [47], which could also be observed in this study. The expression of Nestin gradually decreases from DD0 to DD5 and DD15. However, we still detected a relatively high Nestin expression in some samples at DD25. This indicates that part of the cells remain proliferative neural progenitor cells that do not differentiate to mature neurons.

**Figure 8:**
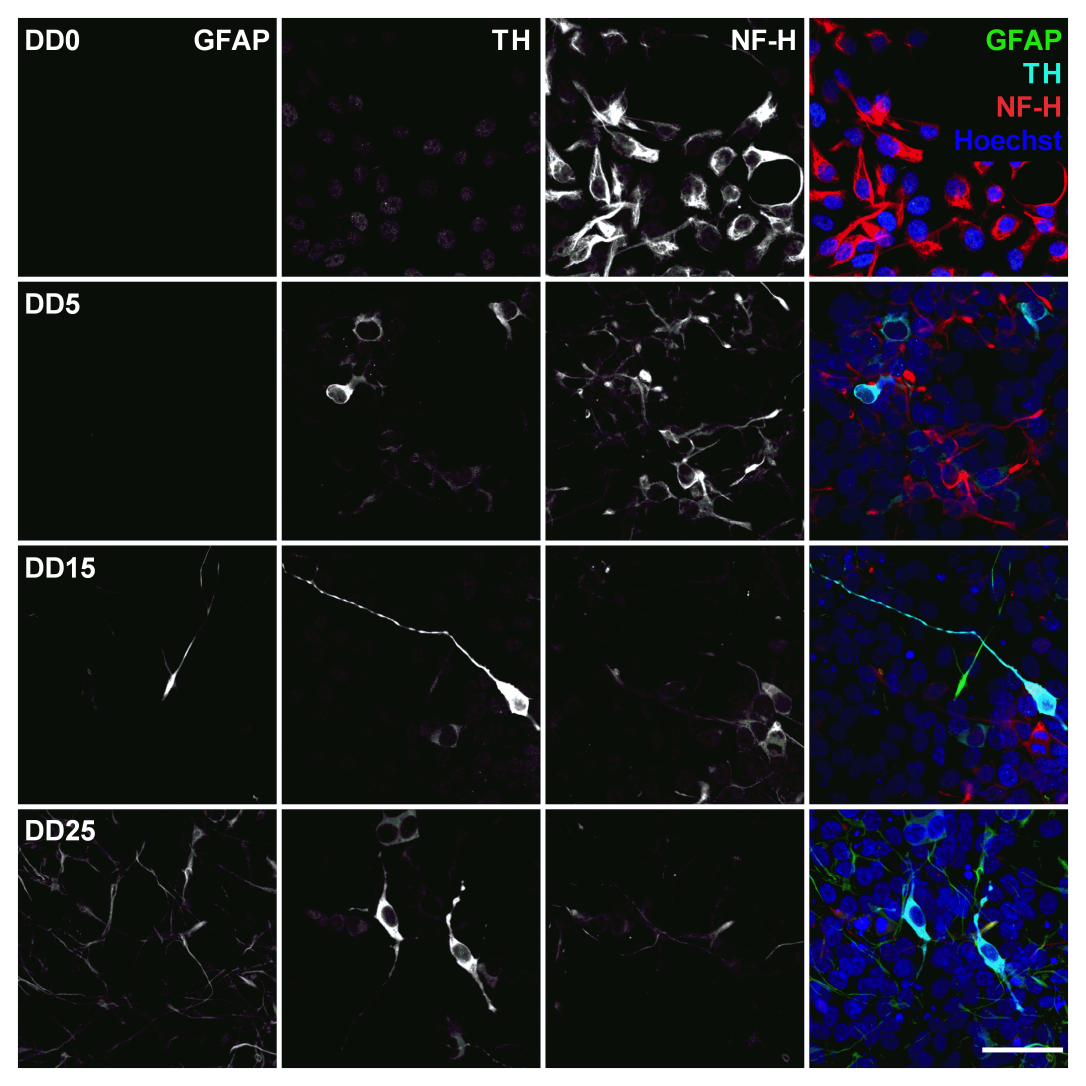
Expression of the astrocyte marker glial fibrillary acidic protein (GFAP), the dopaminergic neuron marker tyrosine hydroxylase (TH), and the neurofilament marker NF-H before (differentiation day DD0) and during neuronal differentiation (DD5, DD15 and DD25) of neural progenitor cells. Nuclei were counterstained with Hoechst. Scale bar corresponds to 50 μ m.

**Figure 9:**
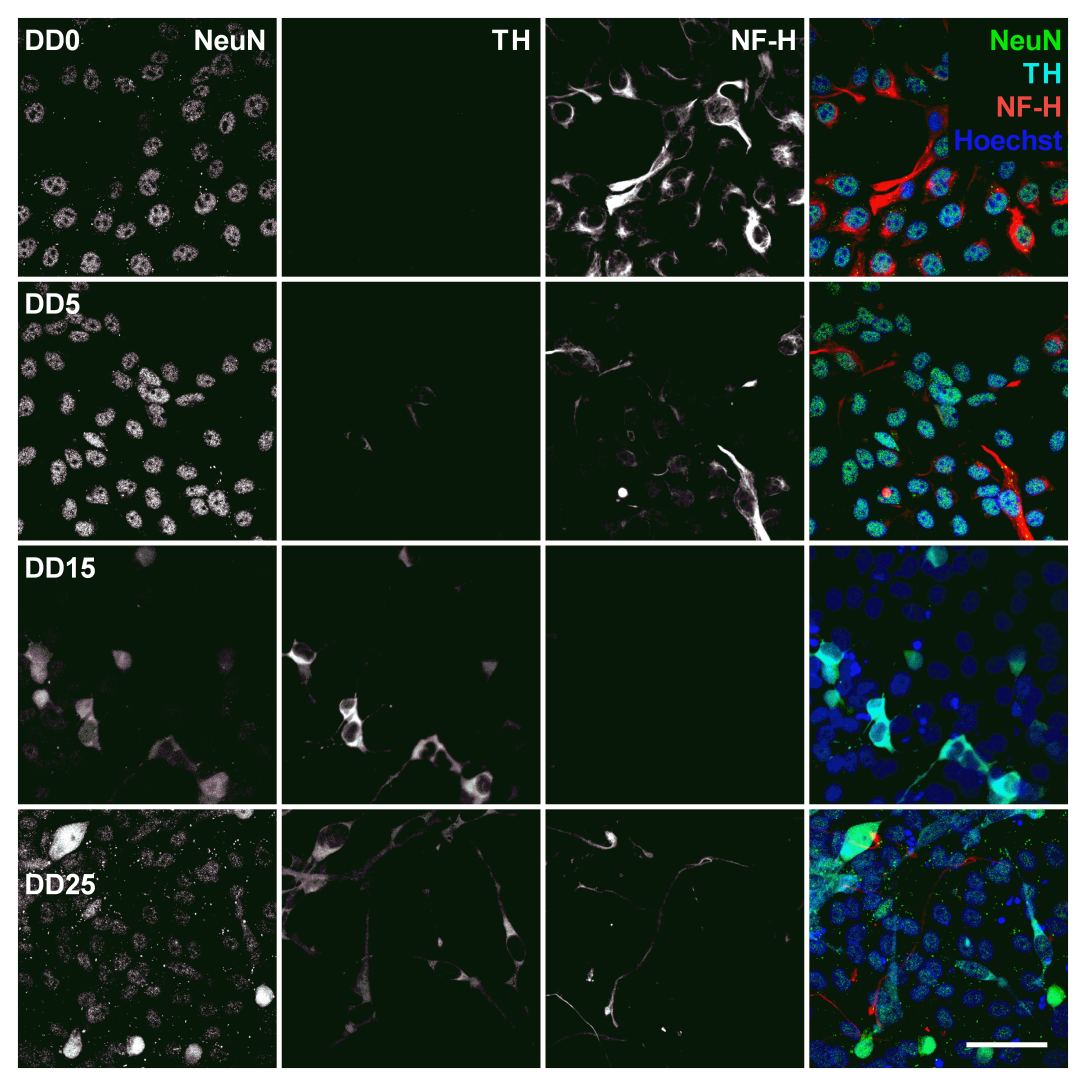
Expression of the neuronal nuclear antigen (NeuN), the dopaminergic neuron marker tyrosine hydroxylase (TH), and the neurofilament marker NF-H before (differentiation day DD0) and during neuronal differentiation (DD5, DD15 and DD25) of neural progenitor cells. Nuclei were counterstained with Hoechst. Scale bar corresponds to 50 μ m.

**Figure 10:**
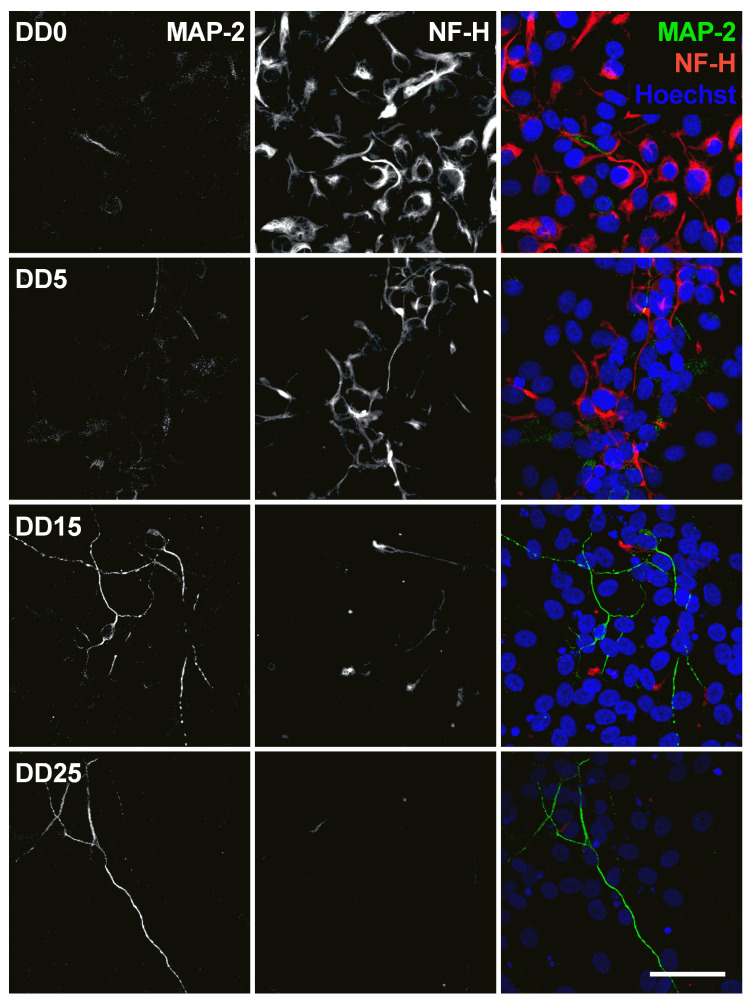
Expression of the neuronal marker microtubule-associated protein 2 (MAP-2) and the neurofilament marker NF-H before (differentiation day DD0) and during neuronal differentiation (DD5, DD15 and DD25) of neural progenitor cells. Nuclei were counterstained with Hoechst. Scale bar corresponds to 50 μ m.

Neurofilament heavy (NF-H) also belongs to the intermediate filament proteins and is expressed in neuronal cell types. NF-H is mainly localized in axons and very little in dendrites and cell bodies of neurons [48]. Surprisingly, NF-H expression could already be detected in the cell bodies of NPCs prior to differentiation and was often co-localized with Nestin expression (see Figure 7, Figure 8, Figure 9 and Figure 10). During differentiation, when the cell morphology changes, NF-H was localized more in the neurites, potentially the axons, than in the cell bodies. However, NF-H expression seemed to decrease, indicating that a high proportion of the NPCs was not differentiating to neurons but presumably to more proliferative glial cells, especially at later time points.

This would be supported by looking at the expression of glial fibrillary acidic protein (GFAP), shown in Figure 8. GFAP is an intermediate filament protein expressed in mature astrocytes [49,50]. No GFAP could be found at DD0 and DD5. Few GFAP-positive cells started to appear at DD15. By DD25, the amount of GFAP-positive presumable astrocytes was outnumbering the amount of neurons. This is typical for a mixed population of neurons and glial cells, as the neurons stop proliferating and even decrease in number when building neuronal networks and maturing, while glial cells remain proliferative and continue to divide in culture [51].

Neuronal Nuclear Antigen (NeuN) is a marker for postmitotic neurons and is not found in immature neural progenitor cells [52]. The protein is localized in the nucleus or perinuclear cytoplasm and is expressed by most neuron subtypes. In this study, however, the basal expression of NeuN inside the nuclei could already be detected in the NPC state at DD0 and DD5, as shown in Figure 9. At DD15 and DD25, several nuclei with strong NeuN expression could be detected, indicating that the culture developed mature neurons, but the majority showed no or only very low NeuN expression.

Tyrosine hydroxylase (TH) is the rate-limiting enzyme involved in dopamine synthesis catalyzing the conversion of the amino acid L-tyrosine to L-DOPA [53,54]. TH is generally used as an early marker for dopaminergic neurons. Tønnesen et al. have shown that neuronal differentiation results predominantly in TH-expressing dopaminergic neurons [55]. In this study, TH expression could already be detected at the earliest differentiation time point investigated, at DD5, as shown in Figure 8 and Figure 9. A high proportion of NeuN-positive cells was also positive for TH, confirming that neuronal differentiation leads predominantly to dopaminergic neurons. However, not all NeuN-positive cells express also TH, indicating that the progenitor cells also differentiate to other neuronal subtypes besides dopaminergic neurons. At later time points, other cell types—potentially glial cells or undifferentiated progenitors—outgrow the TH-positive and the NeuN-positive cells. This results in a reduced ratio of TH-positive presumable dopaminergic cells compared to the total number of cells visible by Hoechst nuclei staining.

The microtubule-associated protein 2 (MAP-2) is essential for the development of early neuronal morphology, especially for development of dendrites, and the maintenance of adult neuronal morphology [56]. MAP-2 is a marker for mature neurons but also for undifferentiated neuroepithelial cells [57]. This was also found in this study, as MAP-2 expression could be detected at all time points and became stronger during longer differentiation at DD15 and DD25 (see Figure 10). However, not many cells were MAP-2-positive, confirming that the majority of cells were not mature neurons. MAP-2 and NF-H are generally not co-localized, which is not surprising as MAP-2 is usually localized in the soma and dendrites [56], while NF-H is usually found in axons [48].

In summary, differentiation of the optogenetically modified human fetal ventral mesencephalic neural progenitor cell line hVM1-Bcl-XL-GFP-ChR2-mCh leads to a diverse mixture of TH-positive presumable dopaminergic neurons, other neuronal subtypes, supporting astrocytes, undifferentiated progenitor cells and possibly other glial cells like oligodendrocytes or microglia, although no markers for other neuron subtypes or glial cells other than astrocytes were investigated. These cells, grown and differentiated on-chip, were used as a model system for all following impedimetric and amperometric measurements to prove the suitability of the developed multisensor platform.

### 3.2. Performance of the Miniaturized Impedance Analyzer Is Comparable to a Desktop Instrument

We compared our PSoC™-based impedance analyzer with the Zurich Instruments MFIA (Zurich Instruments, Zürich, Switzerland), which is a standard desktop impedance analyzer. Impedance was measured between WE and CE using on-chip electrodes (carbon WE, platinum CE and RE). As we can see for measurements on electrodes immersed in buffer solution without cells shown in Figure A9, the PSoC™ impedance analyzer performs very well in the whole frequency range from 10 Hz to 300 kHz. Only the phase angle at very low frequencies shows a small discrepancy between the two instruments. This is not serious, as these frequencies are of little interest for cell measurements, where the region of interest in the kHz range is well behaved in this instrument.

**Figure 11:**
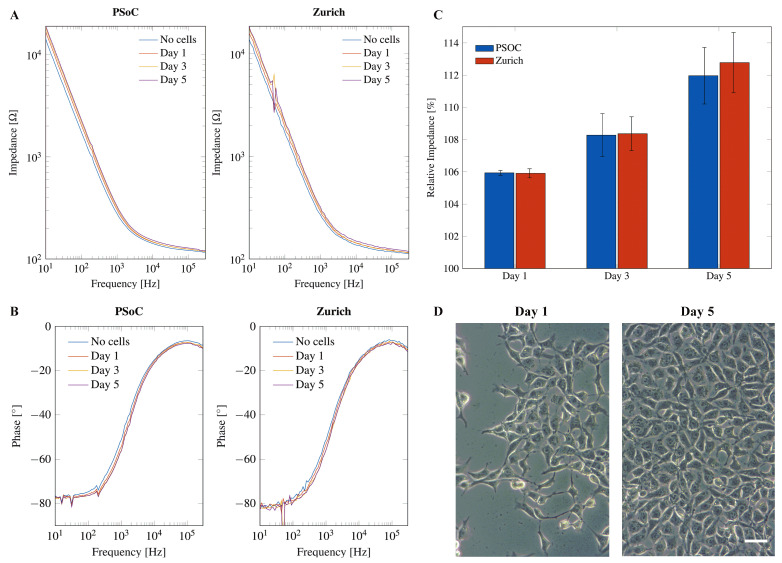
Electrical impedance spectroscopy of human neural progenitor cells (hNPC) for 5 days of cell proliferation in the frequency range from 200 Hz to 300 kHz using both the PSoC™-based impedance analyzer (left) and the Zurich Instruments MFIA (right). (**A**) Impedance magnitude. (**B**) Phase angle. (**C**) Relative impedance (%) for cell growth of hNPCs normalized to the electrode chip without cells (=100%). (**D**) Phase-contrast microscopic images of hNPCs seeded at the same density on cell culture plastic on Day 1 and Day 5 of growth. Scale bar corresponds to 20 μ m.

Neural progenitor cells were seeded on the electrode surface of electrode chips. Impedance was measured during five days of growth on the electrode surface and compared to the impedance of the blank electrode without cells in cell culture medium alone. The medium was replaced by fresh medium 30 min before each impedance measurement to ensure uniform conditions and to avoid effects of the cell culture medium on the impedance. When cells proliferate, the pH value of the cell culture medium will drop and the media composition will change, thereby decreasing the impedance of the SUT. Measurements using the PSoC™-based miniaturized impedance analyzer yield similar results to the Zurich Instruments MFIA for both impedance magnitude (see Figure 11A) and phase angle (see Figure 11B).

If cells grow on the electrode, the impedance increases due to the insulating effect of the cell layer inhibiting the flux of charge between the electrodes [58]. The phase angle shows a tendency to more negative values during cell growth. However, the changes in impedance and phase angle seem very small and require a closer look. A normalization of the impedance values in the range of 200 Hz to 300 kHz on the different days of growth to the impedance without cells results in the relative impedance:

$$relative\;impedance\;\left[\%\right]=\frac{{\left|Z\right|}_{with\;cells}}{{\left|Z\right|}_{without\;cells}}\times 100\%$$

The relative impedance over the frequency range of 200 Hz to 300 kHz was averaged. The average relative impedance values show a clear increase for each day of growth that was similar for the PSoC™-based miniaturized impedance analyzer and the Zurich Instruments MFIA (see Figure 11C). Figure 11D depicts phase contrast microscopy images from NPCs seeded at the same density on transparent cell culture plastic on Day 1 and Day 5 of proliferation.

Overall, the developed miniaturized PSoC™-based impedance analyzer can perform high-fidelity impedance measurements, and its performance is comparable to a desktop impedance analyzer. Cell proliferation can be monitored by the miniaturized instrument with no reduction in quality. A full characterization of the sensing circuit was performed, and published and preliminary tests and cell measurements were reported using an early prototype [37]. Performance measurements in this new iteration can be checked in the Figure A9.

### 3.3. Potentiostat

**Figure A10:**
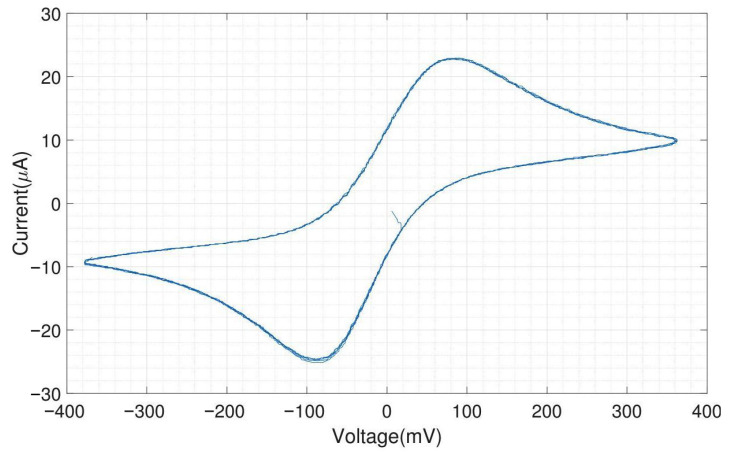
Voltammogramwith on-chip electrodes (carbon WE and CE, gold RE) immersed in 1 mM ferri-/ferrocyanide solution. In this experiment, the scan rate of triangular waveform is 50 $mVs^{-1}$ and the feedback resistor of the TIA is 10 kΩ .

We tested the performance of the headstage potentiostat with different electrochemical solutions. The cyclic voltammogram shown in Figure A10 is obtained by 1 mM potassium ferri-/ferrocyanide solution with on-chip electrodes (carbon WE and CE, gold RE). Ferri-/ferrocyanide are inorganic coordination complexes having a central iron in the oxidation state ${\left[Fe\right]}^{3+}$ (ferricyanide) or ${\left[Fe\right]}^{2+}$ (ferrocyanide). These compounds can be oxidized and reduced to their respective counterparts in a fully reversible manner and can therefore serve as ideal test substances for cyclic voltammetry (CV).

$${\left[Fe{\left(CN\right)}_{6}\right]}^{3-}+e^{-}⇌{\left[Fe{\left(CN\right)}_{6}\right]}^{4-}$$

The CV experiments were performed with a scan rate of triangular waveform of 50 $mVs^{-1}$ and a feedback resistor of the TIA of 10 kΩ . Since the scan rate is low, the capacitive charging current, also called the background current, is small compared to the faradaic current. For 1 mM ferri-/ferrocyanide, the obtained peak cathodic and anodic current were both 25 μ A. The cathodic peak potential was found at 85 mV and the anodic peak potential was found at −85 mV , resulting in a perfectly symmetrical voltammogram and a peak-to-peak separation of 170 mV . For fully reversible redox couples, the ideal predicted peak-to-peak separation is 57/n mV , where n is the number of electrons transferred in the reaction [19].

**Figure A11:**
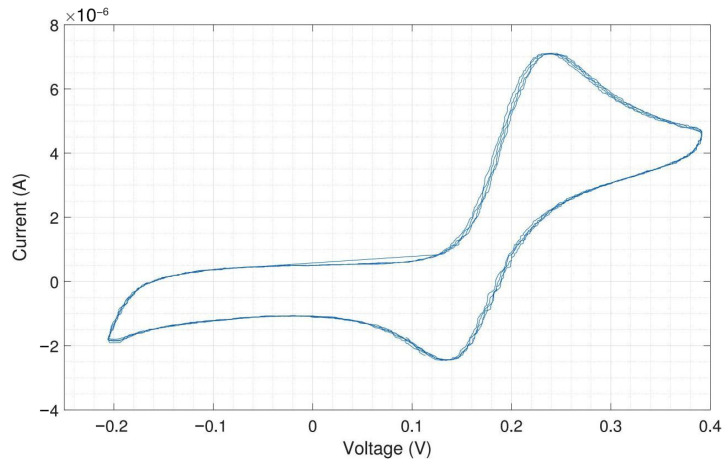
Voltammogramwith on-chip electrodes (carbon WE and CE, gold RE) immersed in 200 μ M dopamine. In this experiment, the scan rate of triangular waveform is 50 $mVs^{-1}$ and the feedback resistor of the TIA is 100 kΩ .

Figure A11 shows the electrochemical measurement results of the headstage potentiostat with the electrode chips immersed in 200 μ M dopamine. The neurotransmitter dopamine is an electroactive molecule, meaning it can be oxidized and reduced by the electric current. Unlike ferri-/ferrocyanide, the oxidation of dopamine is not fully reversible visible in the smaller reduction peak. For dopamine, the obtained peak cathodic current was 6 μ A and the peak anodic current was only 4 μ A. Dopamine is oxidized to dopamine-o-quinone, which diffuses away from the electrode. Therefore, only a part of the oxidized form can be reduced back to dopamine [20]. The cathodic peak potential was 240 mV and the anodic peak potential was 130 mV , resulting in a peak-to-peak separation of 110 mV . Since the oxidation of dopamine to dopamine-o-quinone involves the transfer of two electrons, the ideal peak-to-peak separation would be 28.5 mV .

**Figure A12:**
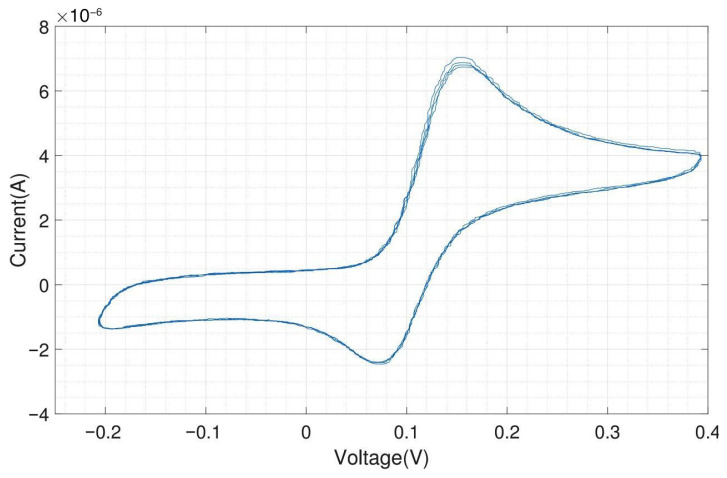
Voltammogramwith on-chip carbon WE, and external CE (Pt) and RE (Ag/AgCl). Electrodes are immersed in 200 μ M dopamine. In this experiment, the scan rate of triangular waveform is 50 $mVs^{-1}$ and the feedback resistor of the TIA is 100 kΩ .

The same experiment was performed with external Ag/AgCl as RE and external Pt as CE, and the result is shown in Figure A12. Silver/silver chloride (Ag/AgCl) is one of the most commonly used REs. As far as the current flow through the RE is zero, the potential drop between the RE and the electrolyte is constant. The amplitudes of the oxidation (6.25 μ A) and reduction peaks (4.25 μ A) are similar to what was obtained with the on-chip RE and CE.

As expected, both experiments provide very similar peak currents while the shift in peak potential, about 80 mV (cathodic peak potential at 155 mV and anodic peak potential at 75 mV ), is due to different materials of RE in these experiments. Platinum is not an ideal reference electrode material and is not so well characterized as the standard Ag/AgCl electrode. But Ag/AgCl electrodes cannot be used for electrode chips on which cells will be cultivated for longer time periods due to the toxicity of silver ions to cells. The external Ag/AgCl electrode also yields a better peak-to-peak separation of around 80 mV instead of 110 mV . For on-chip electrodes, the peak-to-peak separation is almost always higher than for external ones, because there will always be some resistive component in the rather thin contacts on the chip. However, the differences in peak-to-peak separation between on-chip and external electrodes are still relatively low and of no concern.

**Figure A13:**
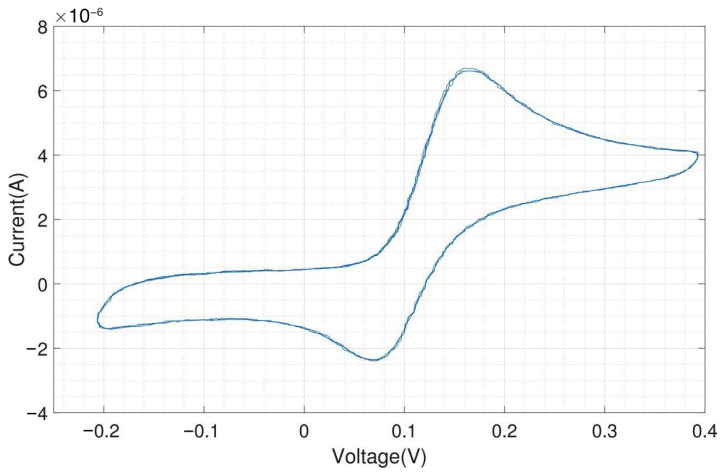
Voltammogramwith on-chip carbon WE and CE, and external RE (Ag/AgCl). Electrodes are immersed in 200 μ M dopamine. In this experiment, the scan rate of triangular waveform is 50 $mVs^{-1}$ and the feedback resistor of the TIA is 100 kΩ .

By using on-chip WE and CE, and external Ag/AgCl, the obtained cyclic voltammogram is shown in Figure A13. The peak currents and location of the peaks (peak cathodic current 6 μ A at 165 mV and peak anodic current 4.1 μ A at 70 mV ) are almost the same as in the voltammogram shown in Figure A12, which was obtained by using an external RE and CE. The peak-to-peak separation of 95 mV is in between the results obtained with the on-chip WE, CE and RE and the results with an external RE and CE.

**Figure A14:**
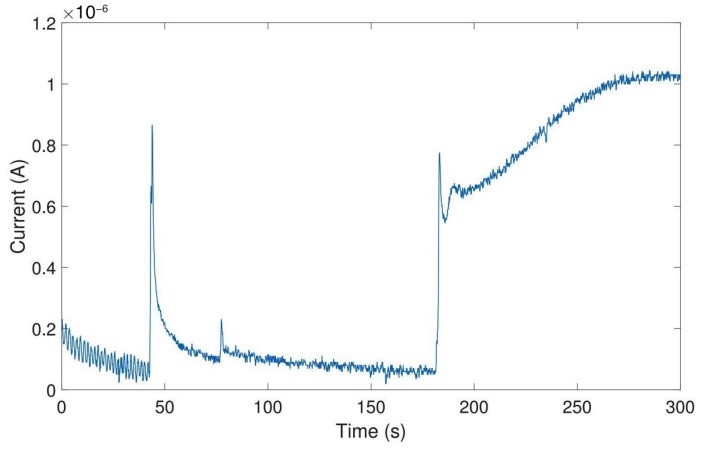
Amperometry measurement with on-chip electrodes (carbon WE and CE, gold RE). Dopamine was added to a PBS solution creating current spikes.

Figure A14 shows measurement results obtained from amperometry with our platform and on-chip electrodes when applying a constant voltage of 250 mV . The peaks in the measured current result from repeated addition of dopamine to the measurement solution with different concentrations. The added dopamine is oxidized at the applied voltage visible as current peaks. The overall results confirm the correct behavior of the platform in amperometry.

#### 3.3.1. The Miniaturized Headstage Potentiostat Is Able to Detect Dopamine Release from Neurons upon Depolarization by Increasing the Extracellular Potassium Concentration

**Figure 12:**
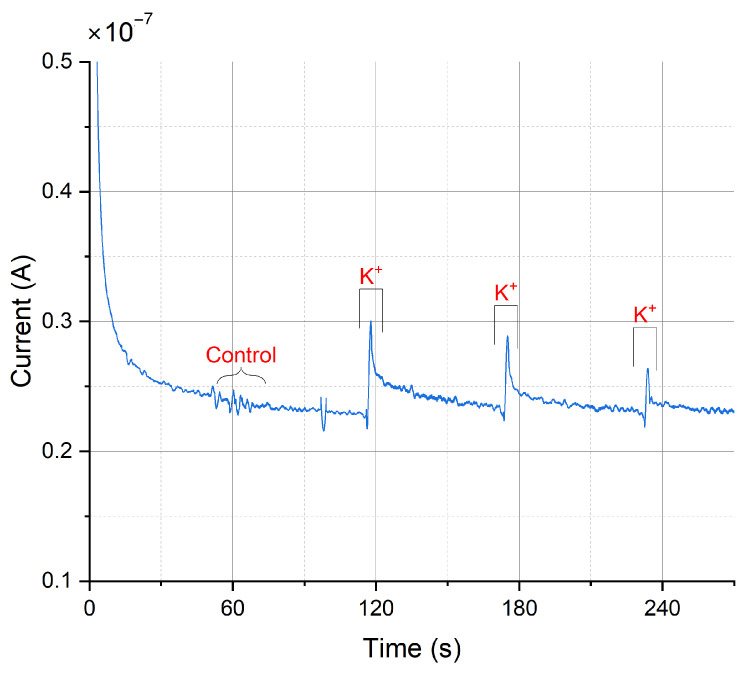
Amperometric detection of dopamine release from differentiated neurons. Current peaks obtained upon depolarization of the neurons by elevating the extracellular K^+^ concentration at 115, 175, and 235 s. At 60 s, the low K^+^ was added as a control.

Dopamine release from populations of adherently growing cells was measured using constant-potential amperometry. Figure 12 shows the obtained results from a chemical stimulation of the cells obtained by injecting a buffer containing a high potassium ion concentration into the wells. Increasing the extracellular K^+^ concentration decreases the gradient between intracellular and extracellular K^+^ concentration. This shifts the membrane potential to more positive values, thus depolarizing the cell, triggering the opening of voltage-gated ion channels and subsequent neurotransmitter release. The constant-potential amperometry result in Figure 12 is obtained by having a feedback resistor of 10 MΩ in the TIA circuit. To improve the sensitivity of the platform, the value of the feedback resistor could be increased. A voltage of 250 mV was applied.

**Figure A15:**
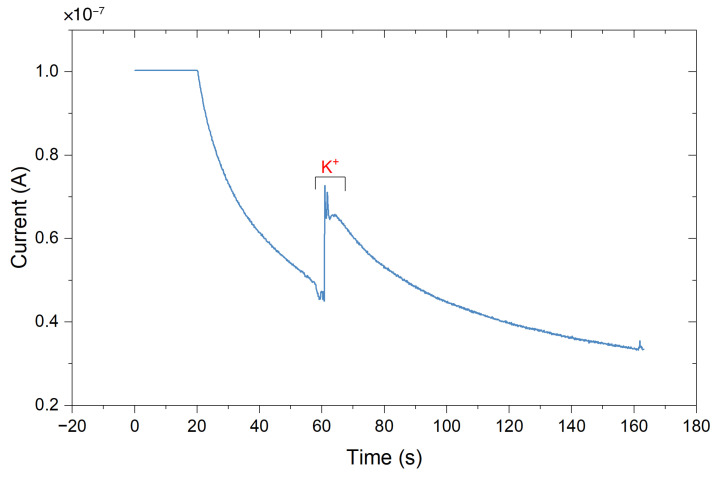
Amperometricdetection of dopamine release from differentiated neurons using a commercial desktop potentiostat (CH Instruments CHI1010A). Current peak obtained upon depolarization of the neurons by elevating the extracellular K^+^ concentration at 60 s.

Currents of 74.4 ± 39.6 nA for carbon electrodes and 72.4 ± 32.3 nA for gold electrodes (mean ± SD, *n* = 3 for carbon electrodes, *n* = 5 for gold electrodes) have been obtained. As a control, a buffer containing a low concentration of K^+^ was injected to the cell culture well. As expected, this created only a minor disturbance but no peak (see Figure 12 at 60 s). In comparison, measurements on single cells (rat pheochromocytoma cell line PC12) reported current pulses of around 6 pA [59]. Therefore, such peaks in the plot are the result from several thousands of cells and exocytotic events, as expected on a large working electrode. In comparison, the peak currents obtained using a commercial desktop potentiostat (CH Instruments CHI1010A) were smaller with an average of 14.2 ± 10.1 nA (mean ± SD, *n* = 3, see Figure A15), but the duration of differentiation was not equal (12–14 days of differentiation for samples measured with commercial potentiostat, 20–31 days of differentiation for samples measured with miniaturized headstage potentiostat).

#### 3.3.2. The Miniaturized Potentiostat Can Also Detect Dopamine Release from Optogenetically Modified Neurons upon Optical Stimulation

**Figure A16:**
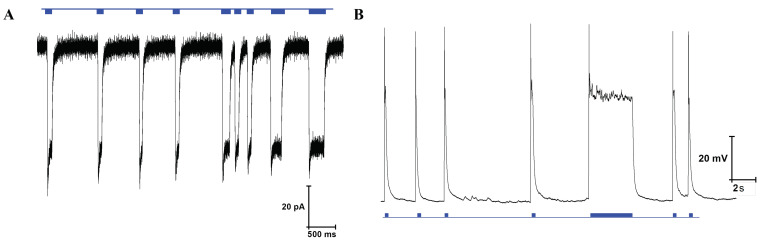
Whole cell patch clamp electrophysiological recording of channelrhodopsin-2 (ChR2) activation by blue light. Representative traces of current (**A**) and voltage (**B**) during voltage clamp and current clamp recordings, respectively.

The cell line used for the experiments was optogenetically modified expressing channelrhodopsin-2 (ChR2) under control of the human synapsin 1 gene promoter. If the progenitor cells are differentiated to neurons, channelrhodopsin-2 will be expressed. This channel is activated by blue light, leading to channel opening, an influx of cations and depolarization of the cell membrane, triggering the release of neurotransmitters [60]. ChR2 activation by blue light was confirmed by whole-cell patch clamp electrophysiological recordings. Representative traces of current and voltage response during stimulation by blue light in voltage clamp and current clamp mode are depicted in Figure A16. Stimulation by blue light leads to an inward current of positively charged cations of around 70 pA in voltage clamp mode or a depolarization of around 80 mV in current clamp mode.

**Figure 13:**
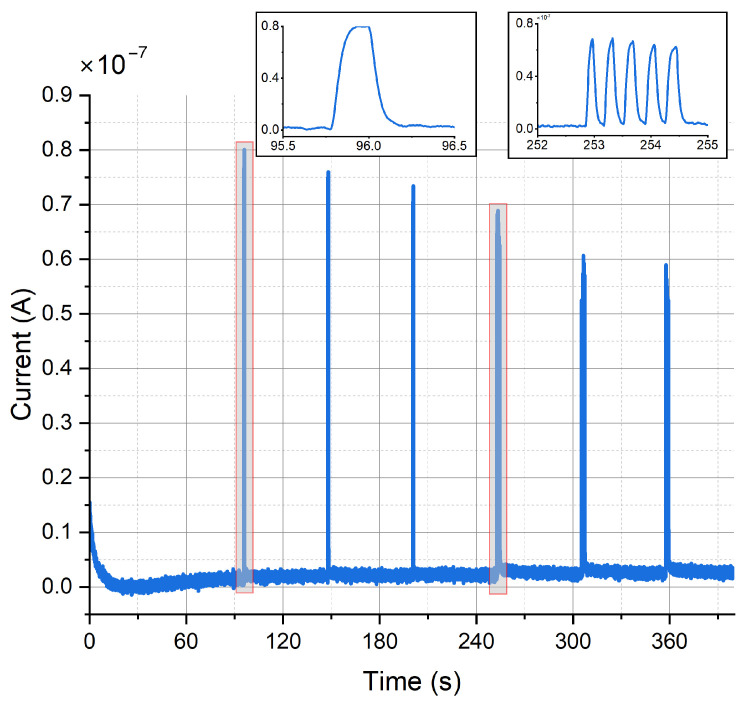
Amperometric detection of dopamine release from differentiated neurons. Current peaks obtained upon optical stimulation of optogenetically modified cells with blue light for single pulses and pulse trains.

Figure 13 shows the constant-potential amperometry experiment result upon optical stimulation with external light source for single and multiple light pulses. The voltage used to perform the experiment was 250 mV . Clear and sharp current peaks were detected. The mean peak amplitude for the first peak was 82.2 ± 5.4 nA for carbon electrodes and 116.5 ± 4.2 nA for gold electrodes (mean ± SD, *n* = 4). Pulse trains consisting of 5 pulses show a clear peak separation. Peak amplitude decreases with repeated depolarization, as the vesicles filled with neurotransmitters become exhausted with every depolarization and need to be replenished.

## 4. Discussion

We present a compact multisensor platform featuring three modules: an impedance analyzer, an optical stimulator and a potentiostat. We show that the platform is able to perform impedance measurements to measure cell growth and that it can be used for optical stimulation and electrochemical measurements (amperometry and cyclic voltammetry). The multisensor platform supports both wired and wireless communication to receive instructions or send measured data. The wireless control is achieved via Bluetooth and is powered by small batteries.

The performed in vitro experiments approve the proper operation of the proposed impedance analyzer and the optoelectrochemical platform. The platform has been coupled with electrode chips and neurons differentiated from optogenetically modified neural progenitor cells on-chip. Upon depolarization of the dopaminergic neurons by increasing the extracellular K^+^ concentration or by optical stimulation of channelrhodopsin-2 (ChR2) by blue light, dopamine is released from the neurons and was detected as current spikes by constant-potential amperometry. The sampling rate of 100 Hz allowed for a clear peak separation of amperometric dopamine detection during optical stimulation both for single pulses as well as for pulse trains of 150 ms pulse duration.

The proposed circuit of the 3-electrode potentiostat for the electrochemical sensing enables amperometry and cyclic voltammetry experiments with a sensitivity of 20 pA . The sensitivity can be optimized based on the electrode dimension with respect to current range, scan rate, etc. The value of the DC voltage used in amperometry to detect dopamine is programmable from −1.8 to +1 V. Therefore, it can be used to detect and study other kinds of electroactive neurotransmitters in addition to dopamine. Similarly, the scan rate and the amplitude of the triangular waveform used for cyclic voltammetry are programmable. Since the feedback capacitor of the TIA used to measure the current is off-chip, it can be modified for different scan rate values in order to optimize the bandwidth and consequently the measurement resolution.

We have used a laser diode as the light source for optical stimulation to improve the power efficiency of the platform. The optical power and stimulation pattern are programmable. Laser stimulation of ChR2 is the standard procedure in optogenetic stimulation experiments. Measurement results demonstrate that the laser diode provides enough optical power at the fiber tip (easily up to 66.82 mW mm^−2^) in order to be used for the stimulation of optogenetically modified dopaminergic cells in a future opto-electric cell implant. However, the electrode chip used in this study, which is shown in Figure A9, has a 12.56 mm^2^ WE area. With this size, the working electrode was about 40 times larger than the WE of the targeted opto-electrical fiber. Therefore, we were not able to detect any signal upon light stimulation with the integrated laser diode due to the large area of the WE. For brain tissue, several sources in the literature refer to around 1–5 mW mm^−2^ as the threshold irradiance for successful stimulation [61]. For in vitro studies, the referred standard value seems to be around 0.3 mW mm^−2^ [62] or close below 1 mW mm^−2^ [63]. However, the threshold activation irradiance is usually used to assess the expression of ChR2 in a certain cell line with stronger expression requiring lower power. In most optogenetic studies, threshold is determined experimentally by trial and error from lower to higher values until a response is measured. The laser stimulator used in our platform was designed with a maximum power delivery of 20 mW. The beam, with a diameter of 1.4 mm at aperture and a divergence of 116 mrad provided at best an irradiance of 0.5 mW mm^−2^. Although this was within the range reported by some studies, it was insufficient for us to see any relevant ChR2 activation. If we assume that an optical power of at least 1 mW mm^−2^ is needed for the activation of ChR2, at least 12.56 mW of optical power at the WE surface would be necessary for these large electrodes. Due to the beam divergence of the LD and the distance between the LD and electrode, it is necessary to provide an optical power even higher than 12.56 mW. Since it is not possible to provide a driving current for the LD that is higher than 40 mA due to the LD driver limitations, our proposed platform can deliver a maximum of 20 mW optical power. Therefore, using the LD incorporated in the platform as a light source to perform experiments on optogenetically modified cells with such a big WE surface did not activate the cells sufficiently. Therefore, an external laser diode with sufficient power was coupled to the system to perform optogenetic experiments. The external laser we used had a beam diameter of 5 mm at aperture and a divergence of 0.5 mrad, providing an irradiance of around 1 mW mm^−2^ at a distance of 50 cm and a 50% ND filter. This proved to be sufficient to perform the experiment and is in agreement with the literature, although it suggests relatively low ChR2 expression. This would be supported by the fact that a polyclonal cell line was used, where not every cell expressed ChR2, and by the degree of differentiation indicated by the cell imaging work performed in this study.

Since the goal of the Training4CRM project was to have a brain-implantable device, which features an electrode chip with much smaller WE area [34,35], our platform can easily provide the required optical power for such devices. The opto-electrical fiber will be integrated with our multisensor headstage platform in a future iteration to perform in vitro and in vivo experiments using the integrated optical stimulator.

Long-term toxic effects of blue light for cells have been shown [64], but alternative approaches [64] such as red light stimulation show that even an optical power of 100–600 mW mm^−2^ is not deleterious for brain tissue and does not induce short-term thermal damage neither at the tissue nor at the cellular level [65]. Several channelrhodopsin variants have been generated, which have different excitation and emission spectra. These techniques might be required for the clinical viability of optogenetics. Although the long-term safety of these techniques for cells and tissues is paramount for clinical applications, one-shot experiments such as the one presented in this work use higher optical power, and the long-term effects to cells were not assessed at this stage and need to be studied.

In the literature, many techniques have been proposed for optical stimulation and electrophysiological recording based on custom chips or discrete components [66,67,68,69,70,71]. Also, many integrated circuits have been proposed to perform electrochemical measurements [72,73,74,75,76,77,78,79,80,81,82]. To the best of our knowledge, there is no custom chip to perform experiments on optogenetically modified dopaminergic cells combining an optical stimulation and an electrochemical detection of the dopamine release. To perform such experiments, bench-top instruments [33,83,84] or potentiostats implemented by off-the shelf components [33] have been used. Recently, an implantable wireless probe for optical stimulation and electrochemical sensing was proposed in [33]. In this work, the authors have fabricated a miniaturized optoelectrochemical probe with embedded blue μ LED and a poly(3,4-ethylenedioxythiophene) polystyrene sulfonate (PEDOT:PSS)-coated diamond film as an electrochemical sensor. To implement a wireless optical stimulation and electrochemical sensing system, a 2.4 GHz RF transceiver with an embedded microcontroller (for remote data communication), a driver chip (to provide a constant current for μ LED), a DAC (to provide a constant or triangular waveform to perform electrochemical measurements), and two pre-amplifiers (to realize the potentiostat circuit) are used. Although they used five commercial chips to implement the system, it is very compact and lightweight. In comparison with our proposed system, the reported headstage in [33] needs fabrication of the μ LED, while the light source in our proposed one is commercially available. Also, the cost of components and system assembly will be lower in our platform, because the reported headstage in [33] has more QFN or similar packages, which requires sophisticated tools for soldering. In addition, we have achieved a resolution as low as 20 pA compared to 100 pA in [33] by using an opamp inside of the PSoC™ chip. Although the system in [33] is more compact compared to our proposed one, we can easily reduce the size of our board according to the electrode chip dimensions.

## 5. Conclusions

In this article, a compact multisensor platform featuring an impedance analyzer, an optical stimulator and a potentiostat for optogenetic experiments is presented. The platform is designed as a headstage for future in vivo experiments. We demonstrated the assessment of cell proliferation by impedance spectroscopy as well as detection of dopamine release from optogenetically modified, differentiated neurons after chemical (elevated extracellular K^+^ concentration) and optical stimulation (activation of ChR2 by blue light).

Future work will include the coupling of the leaky opto-electrical fiber [34,35] to our multisensor headstage platform and performing in vitro and in vivo animal experiments to test the performance in a pre-clinical scenario. For this purpose, it may be necessary to redesign the board—more specifically, the bottom board that hosts the laser diode and electrode. To reduce the size and weight of the system, the board will be fitted to the opto-electrical fiber selected for the in vivo measurements. With the adoption of a machine learning (ML) specialized CPU, we expect to integrate ML-based analysis of the impedance data to automatically assess not only neuronal proliferation but also differentiation [85].

## Figures and Tables

**Figure A3:**
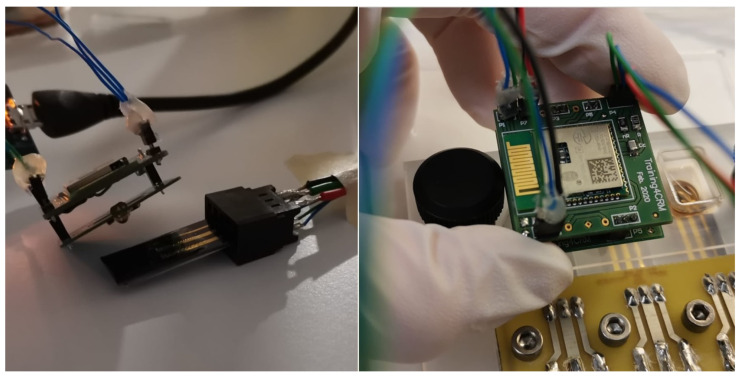
Platform test setup with naked electrode on the **left**, and next to chipholder on the **right**.

**Figure A4:**
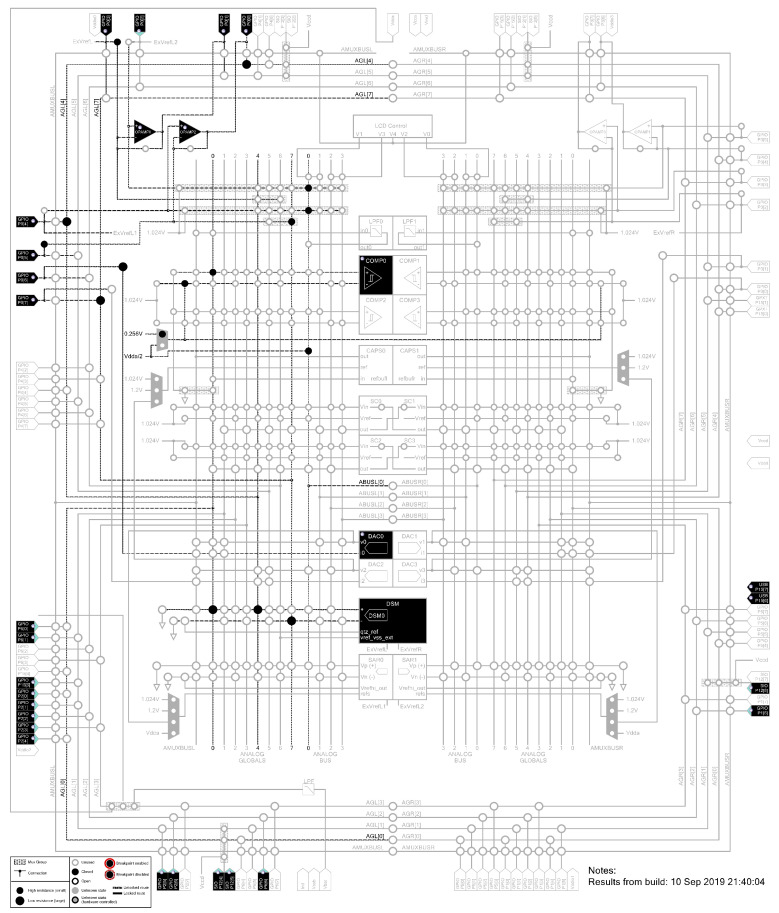
PSoC impedance analyzer complete analog schematic.

## Data Availability

The data presented in this study are available on request from the corresponding author. The data are not publicly available due to protection of intellectual property.

## Abbreviations

The following abbreviations are used in this manuscript:

AC	alternating current
ADC	analog-to-digital converter
BLE	Bluetooth low energy
BM	basic medium
CE	counter electrode
ChR2	channelrhodopsin-2
CRM	cell-based regenerative medicine
CV	cyclic voltammetry
DAC	digital-to-analog converter
DBS	deep brain stimulation
DC	direct current
DD	differentiation day
DM	differentiation medium
FBS	fetal bovine serum
FPGA	field-programmable gate array
GFAP	glial fibrillary acidic protein
GM	growth medium
LD	laser diode
LIA	lock-in amplifier
MAP-2	microtubule-associated protein 2
ML	machine learning
ND	neutral density
NeuN	neuronal nuclear antigen
NF-H	neurofilament heavy
NPC	neural progenitor cell
TH	tyrosine hydroxylase
PBS	phosphate-buffered saline
PCB	printed circuit board
PD	Parkinson’s disease
PSoC™	programmable system on chip
RE	reference electrode
SNR	signal-to-noise ratio
SUT	sample/system under test
TIA	transimpedance amplifier
WE	working electrode

## Appendix A. Additional Figures

### Appendix A.2. Schematics

**Table A1:** Mixer phase state machine LUT.

Input Hex	in1	in0	out2	out1	out0	Output Hex
00	0	0	0	0	1	0x01
01	0	1	0	1	0	0x02
02	1	0	1	1	1	0x07
03	1	1	1	0	0	0x04

**Table A2:** ADC state machine LUT.

Input Hex	in4	in3	in2	in1	in0	out2	out1	out0	Output Hex
00	0	0	0	0	0	0	0	0	0x00
01	0	0	0	0	1	0	1	0	0x02
02	0	0	0	1	0	0	0	0	0x00
03	0	0	0	1	1	0	0	1	0x01
04	0	0	1	0	0	0	1	0	0x02
05	0	0	1	0	1	0	1	0	0x02
06	0	0	1	1	0	0	0	1	0x01
07	0	0	1	1	1	0	0	1	0x01
08	0	1	0	0	0	0	1	0	0x02
09	0	1	0	0	1	0	1	0	0x02
0A	0	1	0	1	0	1	0	0	0x04
0B	0	1	0	1	1	1	0	0	0x04
0C	0	1	1	0	0	0	0	0	0x00
0D	0	1	1	0	1	0	1	1	0x03
0E	0	1	1	1	0	0	0	0	0x00
0F	0	1	1	1	1	0	1	1	0x03
10	1	0	0	0	0	1	0	1	0x05
11	1	0	0	0	1	1	0	1	0x05
12	1	0	0	1	0	1	0	1	0x05
13	1	0	0	1	1	1	0	1	0x05
14	1	0	1	0	0	1	1	0	0x06
15	1	0	1	0	1	1	1	0	0x06
16	1	0	1	1	0	1	1	0	0x06
17	1	0	1	1	1	1	1	0	0x06
18	1	1	0	0	0	1	1	1	0x07
19	1	1	0	0	1	1	1	1	0x07
1A	1	1	0	1	0	1	1	1	0x07
1B	1	1	0	1	1	1	1	1	0x07
1C	1	1	1	0	0	0	1	1	0x03
1D	1	1	1	0	1	0	1	1	0x03
1E	1	1	1	1	0	0	1	1	0x03
1F	1	1	1	1	1	0	1	1	0x03
